# Silencing Myostatin Using In Vivo Self‐Assembled siRNA Protects Against Cancer‐ and Dexamethasone‐Induced Muscle Atrophy

**DOI:** 10.1002/adhm.202502186

**Published:** 2025-10-03

**Authors:** Xin Yin, Azhar Anwar, Jiehao Chen, Qinghao Sun, Likun Zhou, Jie Tang, Jingwei Guo, Linbo Yan, Yongci Chen, Feng Yin, Chen‐Yu Zhang, Zigang Li, Jizheng Ma, Liyuan Sheng, Xi Chen

**Affiliations:** ^1^ PKU‐HKUST ShenZhen‐HongKong Institution Shenzhen Guangdong 518057 China; ^2^ Pingshan Translational Medicine Center, Shenzhen Bay Laboratory Shenzhen Guangdong 518118 China; ^3^ Nanjing Drum Tower Hospital Center of Molecular Diagnostic and Therapy Chinese Academy of Medical Sciences Research Unit of Extracellular RNA State Key Laboratory of Pharmaceutical Biotechnology Jiangsu Engineering Research Center for MicroRNA Biology and Biotechnology NJU Advanced Institute of Life Sciences (NAILS) School of Life Sciences Nanjing University Nanjing Jiangsu 210023 China; ^4^ The Research Center of Military Exercise Science The Army Engineering University of PLA Nanjing Jiangsu 210007 China; ^5^ Tianjin Medical University Cancer Institute and Hospital National Clinical Research Center for Cancer Tianjin's Clinical Research Center for Cancer Key Laboratory of Cancer Prevention and Therapy Tianjin Key Laboratory of Digestive Cancer Tianjin 300060 China; ^6^ State Key Laboratory of Chemical Oncogenomics School of Chemical Biology and Biotechnology Peking University Shenzhen Graduate School Shenzhen 518055 China

**Keywords:** cachexia, dexamethasone, extracellular vesicle, muscle atrophy, myostatin, siRNA

## Abstract

Maintaining skeletal muscle mass is crucial for health, as muscle atrophy caused by drugs, cancer, or aging poses serious risks. However, there are few effective pharmacological interventions targeting muscle atrophy, highlighting the need for new therapeutic strategies. In this study, in vivo self‐assembled siRNA is designed to silence myostatin (MSTN), a key regulator of muscle growth and atrophy, aiming to prevent muscle atrophy. Using synthetic constructs and the host liver as a scaffold, the assembly of MSTN‐siRNA is guided into muscle‐specific peptide MSP‐tagged small extracellular vesicles (sEVs). These MSP‐tagged sEVs selectively deliver MSTN‐siRNA to muscle tissue. Treatment significantly reduces MSTN protein levels in skeletal muscle, promotes muscle mass gain in healthy mice, and protectes skeletal muscles from atrophy in cancer‐ and dexamethasone‐induced muscle atrophy models. Notably, the sEV‐encapsulated MSTN‐siRNA is produced in a nontoxic, nonimmunogenic, and biocompatible manner. This study offers a promising therapeutic approach for muscle atrophy, addressing a key gap in current treatment options and potentially improving outcomes for patients with muscle‐wasting conditions.

## Graphical abstract

This study reports an in vivo self‐assembled siRNA strategy that enables the liver to generate small extracellular vesicles (sEVs) tagged with a muscle‐targeting peptide (MSP) and naturally loaded with myostatin (MSTN)‐siRNA. These MSP‐tagged sEVs are systemically delivered to skeletal muscle, efficiently silence MSTN, promote muscle hypertrophy, and protect against cancer‐ and dexamethasone‐induced muscle atrophy.

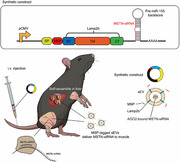

## 1 Introduction

Skeletal muscle atrophy is a pathological condition characterized by the loss of muscle mass and function, which is influenced by a variety of factors such as genetic predispositions, chronic diseases like diabetes and cancer, muscle disuse, drug abuse, and aging.^[^ ^1^ ^]^ This condition severely impairs physical function and mobility, increasing the risk of frailty, falls, and injuries, ultimately reducing the overall quality of life.^[^ ^2^ ^]^ Despite its significant impact, effective clinical treatments for muscle atrophy remain limited, with exercise being the primary intervention. However, compliance with exercise regimens is often challenging for many patients, highlighting the critical need for the development of targeted therapies that can more effectively address the diverse needs of affected individuals.

Myostatin (MSTN), also known as growth differentiation factor 8, is predominantly expressed in muscle tissue and acts as a negative regulator of skeletal muscle mass. MSTN is secreted into circulation, binding to specific receptors to transmit inhibitory signals that limit muscle growth.^[^ ^3^ ^]^ The gene sequence and function of MSTN have remained highly conserved throughout evolution, emphasizing its fundamental role in muscle regulation.^[^ ^4^ ^]^ MSTN gene‐deficient mice exhibit significantly enlarged skeletal muscles, roughly twice the size of normal muscles, making MSTN inhibition an attractive therapeutic strategy for combating skeletal muscle atrophy.^[^ ^3^ ^]^ While preclinical studies in mice have demonstrated that inhibiting or silencing MSTN can prevent muscle atrophy induced by various factors such as cancer‐induced muscle atrophy,^[^ ^5^ ^]^ chronic kidney disease‐induced muscle atrophy,^[^ ^6^ ^]^ and dexamethasone (Dex)‐induced muscle atrophy,^[^ ^7^ ^]^ the outcomes of early clinical trials have been disappointing.^[^ ^4^ ^]^ This highlights the need for novel MSTN‐targeted therapies that can overcome these limitations.

RNA interference () is a potent mechanism for silencing gene expression by targeting messenger RNA (mRNA) and has emerged as a promising therapeutic tool, particularly for genes that are difficult to target with traditional approaches. The evolution of RNAi‐based therapies has seen a transition from the direct injection of naked small interfering RNA (siRNA) molecules to the use of sophisticated delivery vehicles. Among these, vesicle‐encapsulated siRNA drugs have shown significant potential, falling into two main categories: lipid nanoparticles (LNPs) synthesized in vitro and small extracellular vesicles (sEVs) derived from various cell types. Notably, Patisiran, the world's first FDA‐approved siRNA drug, is delivered in vivo using LNP encapsulation.^[^ ^8^ ^]^ Despite recent advances demonstrating that red blood cell platelet‐derived EVs can target muscle tissue, challenges persist with the pre‐assembly of siRNA with carriers or ligands in vitro.^[^ ^9^ ^]^ These challenges include high production and separation costs, low delivery efficiency, potential toxicity, inadequate circulatory stability, and limited tissue accessibility, all of which underscore the need for innovative siRNA delivery systems capable of safely and effectively targeting muscle tissue.^[^ ^10^ ^]^

In response to the challenges associated with delivering siRNA to muscle tissue, we have developed a novel strategy based on synthetic biology that employs the host liver as a tissue chassis for the in vivo self‐assembly of siRNAs (IVSA‐siRNAs). This approach has been shown to effectively deliver siRNAs to various tissues, including the brain,^[^ ^11^, ^12^ ^]^ lung,^[^ ^11^, ^13^ ^]^ and colon.^[^ ^14^ ^]^ However, the delivery of siRNA to muscle tissue remains a significant challenge due to the large mass of skeletal muscle, which constitutes ≈40% of total body mass, complicating systemic delivery via circulation. To address this challenge, we have leveraged the use of a muscle‐specific peptide (MSP, sequence: ASSLNIA) displayed on the membranes of sEVs to enhance delivery efficiency.^[^ ^15^ ^]^ MSP was originally identified through in vivo phage display screening, showing preferential binding to skeletal muscle fibers.^[^ ^15^ ^]^ Subsequent studies have validated the muscle‐targeting capability of MSP across different delivery platforms, including its conjugation to phosphorodiamidate morpholino oligomers for exon‐skipping therapy^[^ ^16^, ^17^ ^]^ and incorporation into adeno‐associated virus (AAV) capsids to enhance muscle tropism.^[^ ^18^ ^]^ Although limited in vivo gene silencing was observed when using exosomes derived from cultured dendritic cells for systemic delivery,^[^ ^19^ ^]^ this limitation was primarily attributed to the low siRNA dosage reaching skeletal muscles, resulting from the large muscle mass and the restricted exosome yield from cell culture, rather than any deficiency in MSP‐mediated targeting.

Building upon these insights, we integrated the MSP into our synthetic constructs, enabling the transcription of MSTN‐siRNA and its subsequent self‐assembly into MSP‐tagged sEVs. This delivery platform achieved effective siRNA targeting across multiple skeletal muscles and was further evaluated for therapeutic efficacy in mouse models of cancer‐ and dexamethasone‐induced muscle atrophy.

## 2 Results

### 2.1 Design of the Synthetic Construct for Generating IVSA‐siRNA Targeting Muscle MSTN

To target MSTN, we designed a synthetic construct that integrates three functional modules: 1) cytomegalovirus (CMV) promoter module: The CMV promoter was employed to drive the transcription of the synthetic construct, including the MSTN‐targeting siRNA and associated components. The choice of the CMV promoter is supported by its strong and ubiquitous expression in various tissues, as established in our previous research;^[^ ^11^ ^]^ 2) Muscle‐targeting module: Targeting specificity was achieved by incorporating an MSP sequence fused to the Lysosome‐associated membrane glycoprotein 2B (Lamp2b) protein, a well‐characterized membrane protein of sEVs. The sequence of this fusion protein was placed downstream of the CMV promoter, allowing for the display of MSP on the sEV membrane. This modification is expected to enhance the targeting efficiency of sEVs toward muscle tissue^[^ ^15^ ^]^; 3) siRNA‐expressing backbone module: To facilitate secretion, the MSTN‐targeting siRNA was embedded into a pre‐miR‐155 backbone, which enhances export via sEVs. And the CMV‐driven pre‐miRNA design was chosen to avoid off‐target effects, with minimal production of the passenger strand. This design was selected to improve the specificity and efficacy of siRNA expression, as indicated by our earlier finding.^[^ ^11^ ^]^ The siRNA‐expressing module was integrated into the synthetic construct downstream of the MSP‐Lamp2b expression cassette.

**Figure 1:**
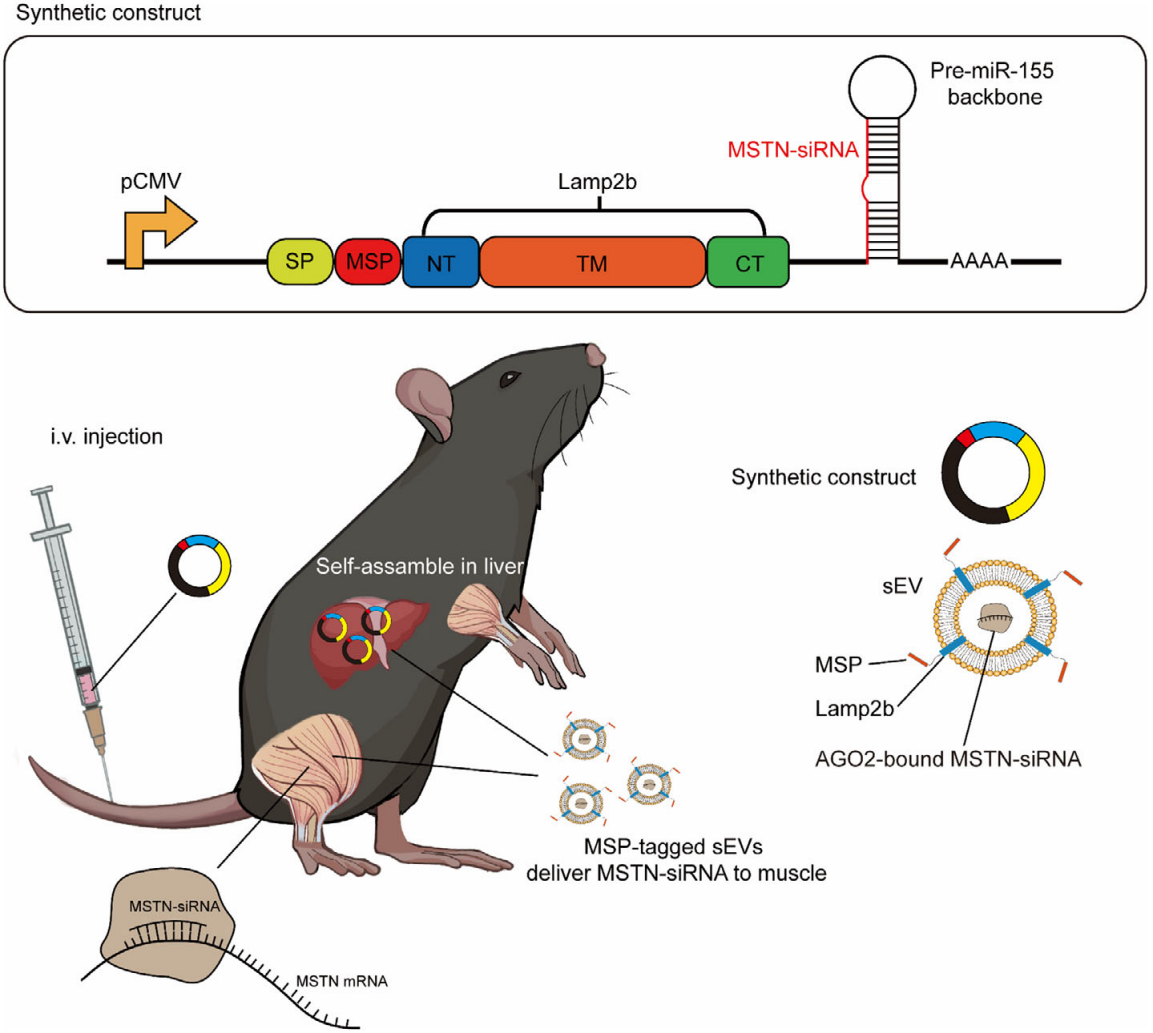
Schematic Representation of the Synthetic Construct Architecture. The synthetic constructs consist of three distinct functional modules: a CMV promoter driving MSTN‐siRNA expression, a muscle‐targeting MSP tag, and a backbone for MSTN‐siRNA expression. Upon integration into a tissue chassis, such as the liver, the CMV promoter initiates MSTN‐siRNA transcription and facilitates its encapsulation into sEVs. Concurrently, the CMV promoter directs the MSP tag to localize on the surface of the sEVs, imparting muscle‐targeting properties. Upon release into the bloodstream, the MSP‐tagged sEVs deliver MSTN‐siRNA specifically to muscle tissues via the sEV circulation system. This targeted delivery mechanism results in MSTN mRNA degradation and reduced protein expression.

The resulting construct, named CMV‐MSP‐siR^MSTN^, is expected to function as follows: Upon intravenous injection and uptake by liver cells, the CMV promoter drives the expression of both the MSP‐Lamp2b fusion protein and MSTN‐siRNA in the liver. The MSP‐tagged sEVs loaded with MSTN‐siRNA are then secreted into the bloodstream, where the MSP facilitates targeted delivery of MSTN‐siRNA‐encapsulating sEVs to muscle tissue, enhancing the therapeutic potential of the MSTN‐siRNA on muscle atrophy (**Figure** **1**).

### 2.2 In Vitro Characterization of the Synthetic Constructs for Silencing MSTN

We designed four CMV‐MSP‐siR^MSTN^ constructs targeting different sites within the open reading frame (ORF) of the MSTN gene to optimize silencing potency. In addition, we used a CMV‐directed synthetic construct co‐expressing an MSP‐Lamp2b fusion protein and a scrambled RNA (CMV‐MSP‐scrR) as a control. To validate the functionality of the CMV‐MSP‐siR^MSTN^ constructs in vitro, we transfected HEK293T cells with a plasmid expressing the MSTN gene tagged with GFP (pcDNA‐MSTN‐GFP). Subsequently, the four CMV‐MSP‐siR^MSTN^ constructs and the control CMV‐MSP‐scrR construct were transfected into these cells (Figure S1A, Supporting Information). All CMV‐MSP‐siR^MSTN^ constructs were found to efficiently produce mature MSTN‐siRNAs (Figure S1B, Supporting Information), leading to significant inhibition of both MSTN protein and mRNA expression in HEK293T cells, with the CMV‐MSP‐siR^MSTN^‐4 construct exhibiting the highest interference efficiency (Figure S1C–E, Supporting Information). This experiment was replicated in C2C12 myotubes, where similar results were observed. All constructs significantly reduced MSTN protein and mRNA levels, and again, the CMV‐MSP‐siR^MSTN^‐4 construct demonstrated superior silencing efficiency (Figure S1F–I, Supporting Information). To further assess the biological impact of MSTN inhibition, the CMV‐MSP‐siR^MSTN^‐4 construct was transfected into C2C12 myotubes to evaluate its effect on myotube diameters. Confocal immunofluorescence imaging and subsequent quantification revealed a significant increase in mean myotube diameter in the cells treated with CMV‐MSP‐siR^MSTN^‐4 construct compared to the mock cells and the cells treated with CMV‐MSP‐scrR (Figure S1J, Supporting Information). Overall, these results demonstrate that the synthetic constructs, particularly CMV‐MSP‐siR^MSTN^‐4, effectively produce mature, functional MSTN‐siRNA in vitro, resulting in potent inhibition of MSTN expression and subsequent modulation of myotube size.

### 2.3 Ex Vivo Characterization of the Synthetic Construct for Producing sEV‐Encapsulated MSTN‐siRNA

**Figure 2:**
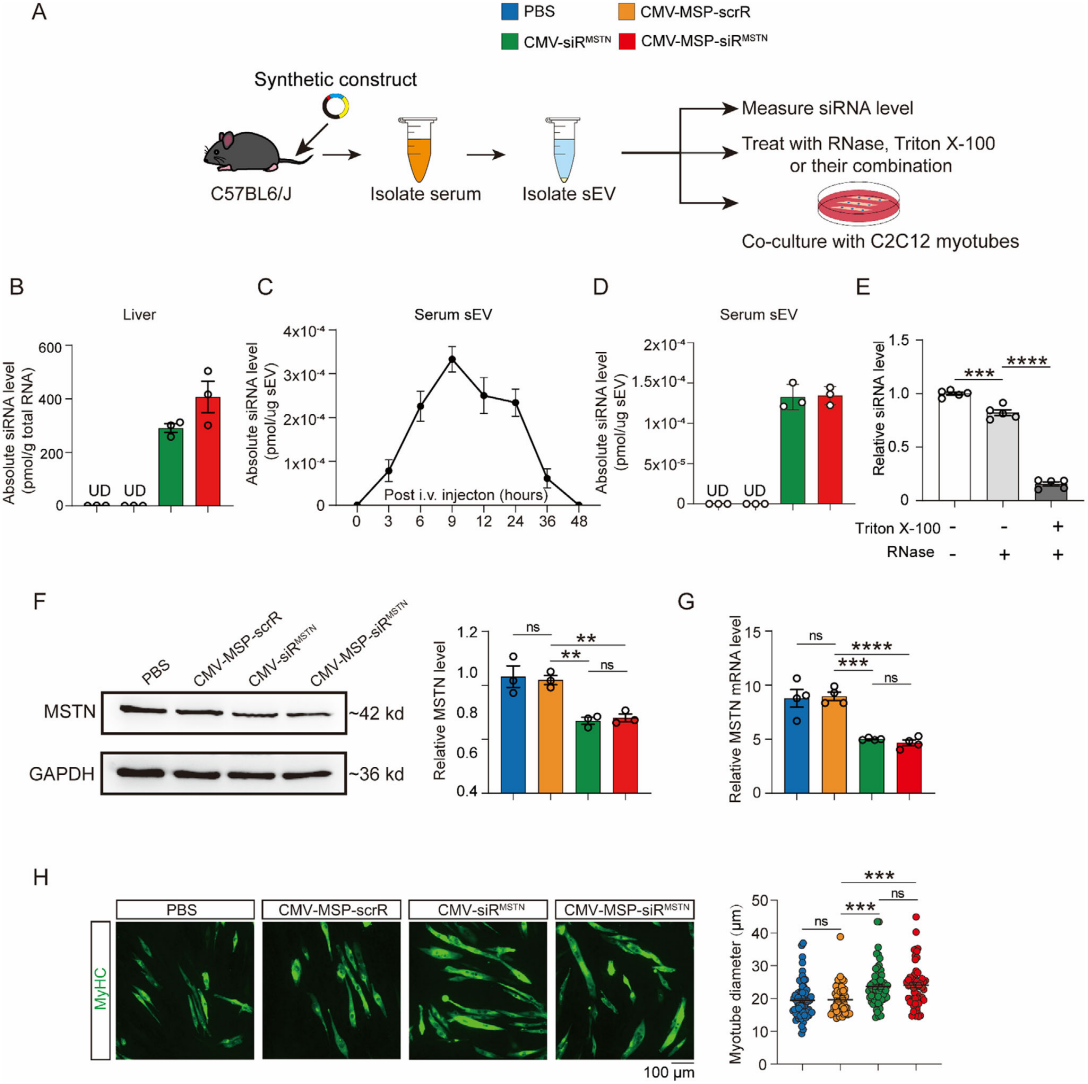
Characterization of Self‐Assembled MSTN‐siRNA in an Ex Vivo Model. A) Schematic of the experimental design. C57BL/6J mice were intravenously injected with PBS or 5 mg kg^−1^ CMV‐MSP‐scrR, CMV‐siR^MSTN^, or CMV‐MSP‐siR^MSTN^ every 2 days for a total of seven injections. The sEVs were purified from mouse serum and added to the culture medium of C2C12 myotubes. After 48 h, MSTN expression levels and myotube diameters in C2C12 myotubes were assessed. B) Quantitative RT‐PCR analysis of MSTN‐siRNA levels in the liver, conducted 6 h after the final injection (n = 3). C) Quantitative RT‐PCR analysis of MSTN‐siRNA kinetics in serum sEVs at different time points (n = 3). D) Isolated sEVs with high MSTN‐siRNA levels (9 h after the final injection) were co‐cultured with C2C12 myotubes, and quantitative RT‐PCR analysis of MSTN‐siRNA levels in purified sEVs was performed (n = 3). E) sEVs from the serum of mice injected with CMV‐MSP‐siR^MSTN^ construct for a total of 7 times were extracted through differential centrifugation. After treatment with or without 1% Triton X‐100, sEVs were digested with RNase. Then Trizol reagent was added to extract total RNA followed by quantitative RT–PCR detection of MSTN‐siRNA. Equivalent amount of RNA extracted from sEVs without any treatment was used as a control (n = 5). F) Western blot analysis of MSTN protein levels in C2C12 myotubes after 48 h’ incubation with serum sEVs. Representative Western blot (left panel) and densitometric analysis (right panel) are shown (n = 3). G) Quantitative RT‐PCR analysis of MSTN mRNA levels in C2C12 myotubes after 48 h’ incubation with serum sEVs (n = 4). H) Representative images of immunofluorescence staining for MyHC protein (left panel) and corresponding diameter statistics (right panel). Scatter plots analyze more than 50 myotubes, with average values shown. Scale bar: 100 µm. Data are presented as mean ± SEM. Statistical significance was determined using one‐way ANOVA followed by Bonferroni's multiple comparisons test for panels E, F, and G. ^**^ *p* < 0.01; ^***^ *p* < 0.001; ^****^ *p* < 0.0001; UD = Undetectable. ns = not significant.

Given the complexity of the in vivo environment, we turned to an ex vivo model to assess whether the synthetic construct could autonomously assemble muscle‐targeted sEVs encapsulating MSTN‐siRNA. Specifically, we focused on the production, assembly, and release of MSTN‐siRNA within endogenous sEVs. To achieve this, C57BL/6J mice were intravenously injected with PBS, CMV‐MSP‐scrR, CMV‐siR^MSTN^, or CMV‐MSP‐siR^MSTN^ every two days for a total of seven injections. Afterward, the mice were sacrificed, and the circulating sEVs were isolated from the serum for siRNA level measurement or co‐culture with C2C12 myotubes (**Figure** **2**A).

Subsequently, we employed nanoparticle tracking analysis (NTA) and transmission electron microscopy (TEM) to characterize these isolated sEVs and detected canonical sEV markers on these sEVs. Our NTA results revealed a consistent size distribution (≈124 nm) and comparable concentrations across all groups (Figure S2A, Supporting Information). TEM confirmed the presence of double‐layered membrane structures and the typical cup‐shaped morphology of sEVs (Figure S2B, Supporting Information). Additionally, Western blotting detected the sEV markers Alix, CD9, and CD63 in the isolated sEVs (Figure S2C, Supporting Information). These results clearly demonstrated that the synthetic construct does not alter the characteristics of endogenous sEVs.

Our previous study suggests that the host liver can serve as a scaffold for guiding siRNA self‐assembly into peptide‐tagged sEVs.^[^ ^11^ ^]^ Herein, we examined the accumulation of MSTN‐siRNA in the liver. Our results showed that both CMV‐siR^MSTN^ and CMV‐MSP‐siR^MSTN^ resulted in similar levels of MSTN‐siRNA in the liver 6 h post‐injection (Figure 2B). Additionally, we observed the kinetics of MSTN‐siRNA in serum sEVs, with a peak at 9 h post‐injection that returned to baseline by 48 h (Figure 2C). These findings indicate that the host liver can efficiently take up the injected synthetic constructs, assemble MSTN‐siRNA‐encapsulated sEVs, and release them into the blood circulation.

To assess the knockdown effect of the IVSA‐siRNA targeting MSTN, we co‐cultured the isolated sEVs with C2C12 myotubes. The sEVs, isolated from mouse serum 9 h after the injection of CMV‐siR^MSTN^ or CMV‐MSP‐siR^MSTN^, had equivalent levels of MSTN‐siRNA, consistent with the observed siRNA kinetics (Figure 2D). To investigate the stability of siRNA generated from the CMV‐MSP‐siR^MSTN^ construct, we performed RNase protection assays using serum sEVs isolated from mice injected with CMV‐MSP‐siR^MSTN^. MSTN‐siRNA levels remained stable after RNase treatment but were substantially degraded when sEV membranes were disrupted by Triton X‐100, indicating that the lipid bilayer of sEVs effectively protects siRNAs from RNase‐mediated degradation (Figure 2E). Furthermore, co‐culture of these sEVs with C2C12 myotubes efficiently resulted in the knockdown of MSTN protein and mRNA levels (Figure 2F,G) and increased myotube diameters (Figure 2H).

### 2.4 In Vivo Tracking the Delivery of sEV‐Encapsulated MSTN‐siRNA to Skeletal Muscle

**Figure 3:**
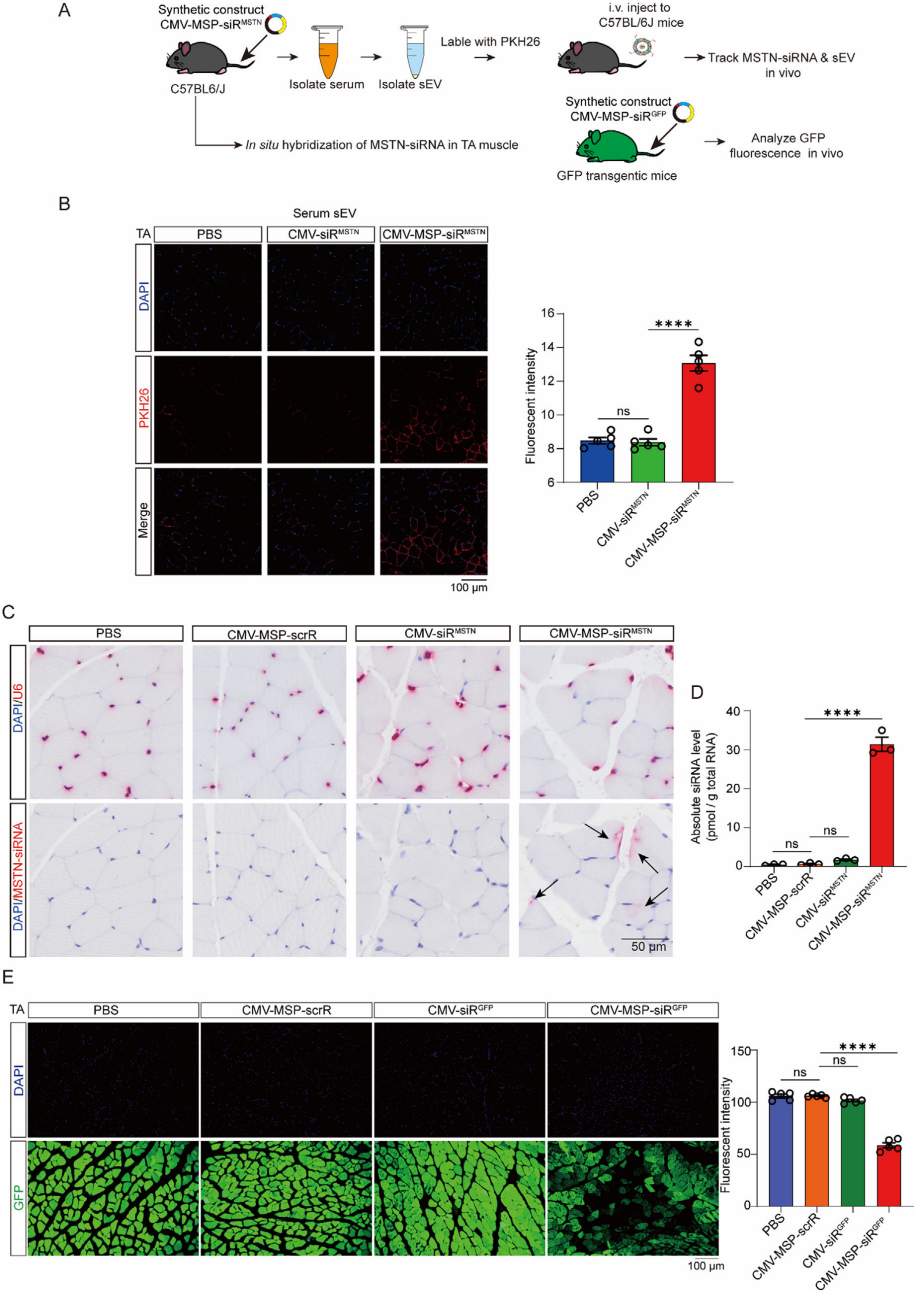
Tracking and Visualization of the Delivery of Self‐Assembled MSTN‐siRNA to Skeletal Muscle. A) Schematic diagram of the experimental design for tracking and visualization. For sEV tracking, C57BL/6J mice were intravenously injected with PBS, CMV‐siR^MSTN^, or CMV‐MSP‐siR^MSTN^ every 2 days for a total of seven injections. 9 h after the final injection, serum sEVs were purified, labeled with PKH26 dye, and injected via the tail vein into new mice to detect sEV fluorescence distribution in the TA muscle. For MSTN‐siRNA visualization, C57BL/6J mice were injected with PBS, CMV‐MSP‐scrR, CMV‐siR^MSTN^, or CMV‐MSP‐siR^MSTN^ every 2 days for a total of seven injections. Twelve hours after the final injection, TA muscle samples were collected to detect MSTN‐siRNA. For in vivo suppression of GFP fluorescence by self‐assembled GFP siRNA, GFP‐transgenic mice were injected with PBS, CMV‐MSP‐scrR, CMV‐siR^GFP^, or CMV‐MSP‐siR^GFP^ constructs every 2 days for seven times. Twelve hours after the final injection, mice were sacrificed, and GFP fluorescence levels were assessed in frozen sections of TA muscle. B) Representative images showing the distribution of fluorescent sEVs 24 h after injection (left panel) and the corresponding quantification (right panel) (n = 5). Scale bar = 100 µm. C) Positive in situ hybridization signals in TA muscle are shown in red, with DAPI‐stained nuclei in blue. Scale bar = 50 µm. D) Absolute levels of MSTN‐siRNA in TA muscle measured using quantitative RT‐PCR (n = 3). E) Representative GFP fluorescence images (left panel), where positive GFP signals are shown in green with DAPI‐stained nuclei in blue, and the corresponding quantification (right panet) (n = 5). Scale bar = 100 µm. Significance was determined using one‐way ANOVA followed by Bonferroni's multiple comparisons test for panels B, D, and E. ^****^ *p* < 0.0001; ns = not significant.

To demonstrate the in vivo delivery and presentation of MSTN‐siRNA by MSP‐tagged sEVs to skeletal muscle, we conducted in vivo tracing of serum sEVs following synthetic construct injection and utilized miRNAscope technology for in situ hybridization to track MSTN‐siRNA within skeletal muscle. C57BL/6J mice were intravenously injected with PBS, CMV‐siR^MSTN^, or CMV‐MSP‐siR^MSTN^ every two days for a total of seven injections.9 h after the final injection, serum sEVs were purified, labeled with PKH26 dye, and injected via the tail vein into new mice to detect the distribution of sEV fluorescence and siRNA signals within skeletal muscle (**Figure** **3**A). A significant accumulation of PKH26 fluorescence (red) was observed in the quadriceps (Qua), gastrocnemius (Gas), and tibialis anterior (TA) muscles of recipient mice injected with CMV‐MSP‐siR^MSTN^, compared to other groups (Figure 3B; Figure S3, Supporting Information), indicating that the MSP‐Lamp2b fusion protein effectively confers skeletal muscle‐targeting capability to the engineered sEVs. Subsequently, miRNAscope technology was employed to detect the distribution of MSTN‐siRNA within TA muscle. SiRNA signals (indicated by black arrows) were detected only in the skeletal muscle of mice injected with CMV‐MSP‐siR^MSTN^ (Figure 3C). Moreover, we measured the expression levels of MSTN‐siRNA in the TA muscle following systemic administration and found that the concentration of MSTN‐siRNA in TA muscle reached ≈30 pmol g^−1^ total RNA, whereas MSTN‐siRNA was barely detectable in the other groups (Figure 3D). These results confirm the successful delivery of sEV‐encapsulated MSTN‐siRNA to skeletal muscle.

To demonstrate that the delivered MSTN‐siRNA can effectively regulate target gene expression within skeletal muscle, we utilized transgenic mice ubiquitously expressing GFP to evaluate the muscle‐targeting capability of IVSA‐siRNA. We developed a CMV‐directed synthetic construct expressing an GFP‐silencing siRNA, either with or without an MSP‐Lamp2b fusion protein (denoted as CMV‐MSP‐siR^GFP^ or CMV‐siR^GFP^). The GFP transgenic mice were intravenously injected with PBS, CMV‐MSP‐scrR, CMV‐siR^GFP^, or CMV‐MSP‐siR^GFP^ every two days for a total of seven injections. The mice were then sacrificed to measure GFP fluorescence in the liver, lung, kidney, heart, and several skeletal muscles. Our results revealed a significant reduction in GFP fluorescence in the liver, lung, and kidney of GFP transgenic mice injected with either CMV‐siR^GFP^ or CMV‐MSP‐siR^GFP^, compared to the controls injected with PBS or CMV‐MSP‐scrR (Figure S4C–F, Supporting Information). Notably, a significant reduction in GFP fluorescence was observed in the Qua, Gas and TA muscles of mice injected with CMV‐MSP‐siR^GFP^, compared to other groups (Figure 3E; Figure S4A–B, Supporting Information). In addition, we evaluated GFP siRNA delivered via LNPs through both tail vein and local intramuscular injection. While systemic LNP delivery had no observable impact on GFP expression in TA (Figure S5A, Supporting Information), local administration resulted in robust knockdown at the injection site (Figure S5B, Supporting Information), however, GFP fluorescence in the contralateral TA muscle without LNP injection remained unaffected. These findings demonstrate that skeletal muscle‐targeted IVSA‐siRNA enables efficient and specific siRNA delivery to multiple skeletal muscles via systemic administration of the synthetic construct, and thus represents a clinically relevant alternative to locally administered siRNA delivery methods.

### 2.5 Evaluation of the Effects of IVSA‐siRNA Targeting MSTN on Modulation of Muscle Mass

**Figure 4:**
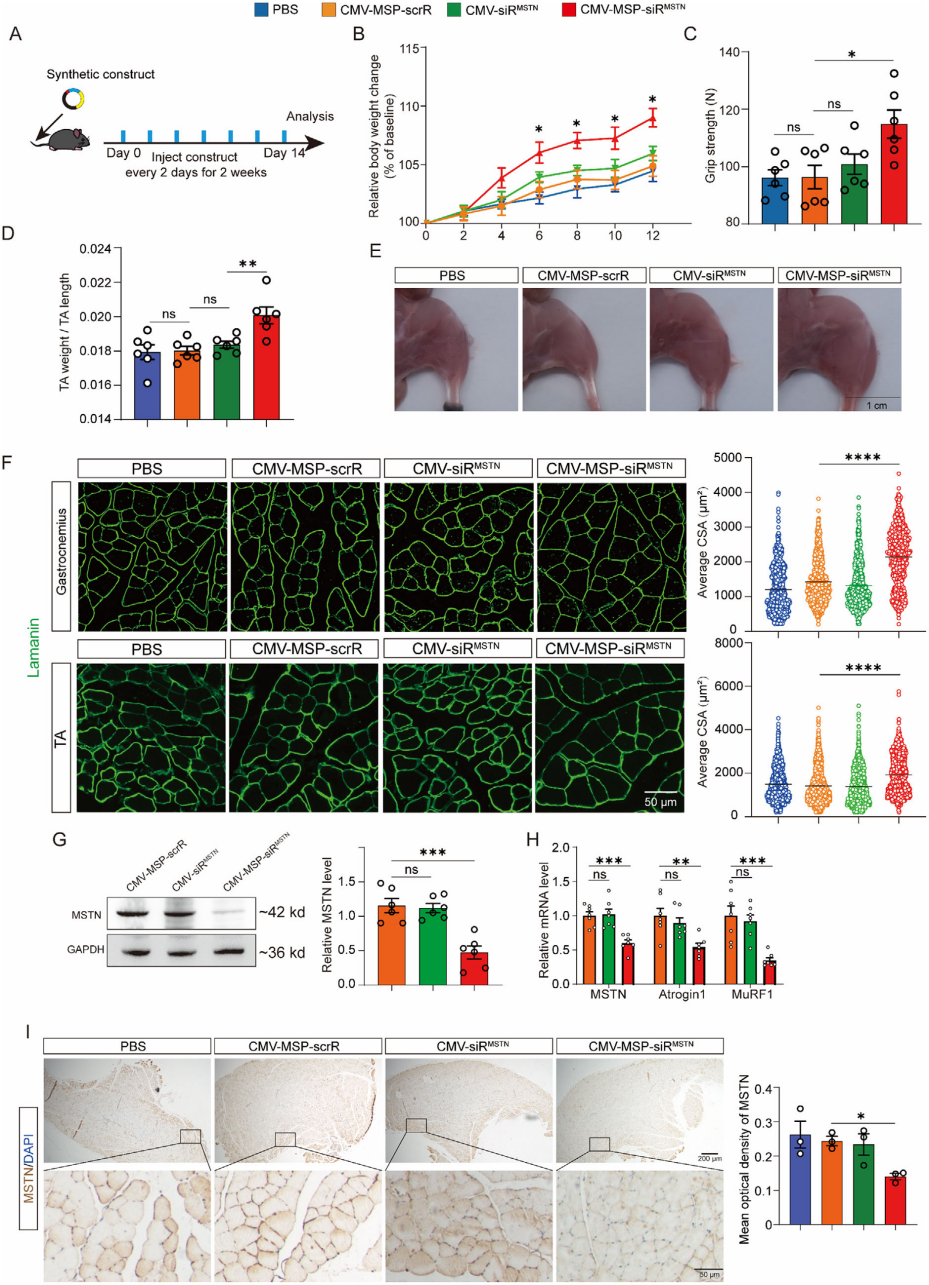
Modulation of Skeletal Muscle Mass in Healthy Wild‐Type Mice by In Vivo Self‐Assembled MSTN‐siRNA. A) Schematic of the experimental design. Synthetic constructs were administered to wild‐type mice every 2 days for a total of seven injections to evaluate muscle mass. B) Body weight curves (n = 7). C) Muscle function assessed using grip strength measurement (n = 6). D) Determination of TA muscle weight (n = 6). E) Representative images of hindlimbs from each group. Scale bar = 1 cm. F) Laminin immunofluorescent staining of cross‐sections of gastrocnemius and TA muscles (left panel), with corresponding cross‐sectional area statistics. The scatter plot analyzes over 500 muscle fibers from 5 mice, with average values shown. Scale bar = 50 µm. G) Western blot analysis of MSTN protein levels in TA muscles. Representative Western blot (left panel) and densitometric analysis (right panel) are shown (n = 5). H) Quantitative RT‐PCR analysis of MSTN, Atrogin1, and MuRF1 mRNA levels in TA muscles (n = 7). (I) Representative images of IHC staining for MSTN in TA muscle sections and corresponding quantification (n = 3). Scale bar = 200 µm (upper panel) and 50 µm (lower panel). Data are presented as mean ± SEM. Significance was determined using one‐way ANOVA followed by Bonferroni's multiple comparisons test for panels B‐D and F‐I. ^*^ *p* < 0.05; ^**^ *p* < 0.01; ^***^ *p* < 0.001; ^****^ *p* < 0.0001; ns = not significant.

To evaluate the impact of synthetic constructs on muscle mass regulation, we administered 5 mg kg^−1^ of the constructs to wild‐type male C57BL/6J mice every other day for two weeks (**Figure** **4**A). Compared to control groups treated with CMV‐MSP‐scrR or CMV‐siR^MSTN^, the group treated with CMV‐MSP‐siR^MSTN^ displayed a significant increase in body weight, grip strength, and the TA muscle weight‐to‐TA length ratio (Figure 4B–D). Skeletal muscle modulation was further evident in the enlarged hindlimb muscle sizes in mice treated with the CMV‐MSP‐siR^MSTN^ construct (Figure 4E). Immunofluorescence staining for Laminin confirmed this modulation by showing significantly larger cross‐sectional areas (CSAs) in both the gastrocnemius and TA muscles (Figure 4F). Additionally, Western blot analysis revealed a substantial reduction in MSTN protein levels in the TA muscle following treatment with the CMV‐MSP‐siR^MSTN^ construct compared to the control groups (Figure 4G). This decrease was consistent with a significant reduction in MSTN mRNA levels and the expression of MSTN's downstream effectors, E3 ubiquitin ligases MuRF1 and Atrogin1 (Figure 4H).^[^ ^20^ ^]^ Furthermore, immunohistochemical staining confirmed a significant reduction in MSTN levels within TA muscle sections of mice treated with the CMV‐MSP‐siR^MSTN^ construct (Figure 4I). These results suggest that IVSA‐siRNA targeting MSTN can influence muscle mass regulation, providing a basis for further exploration in atrophy models.

### 2.6 Evaluation of the Therapeutic Effects of IVSA‐siRNA Targeting MSTN in Cancer‐Induced Muscle Atrophy Model

Muscle atrophy, a common occurrence associated with skeletal muscle tissue, is linked to numerous disease states. This leads to frailty and inhibits patients’ mobility, resulting in a decline in their quality of life.^[^ ^21^ ^]^ Furthermore, muscle loss is an unfavorable indicator of the progression of underlying diseases. For example, cancer‐associated cachexia, a debilitating condition characterized by both adipose and muscle atrophy, compromise responses to anticancer therapy and inevitably shorten survival.^[^ ^22^ ^]^ Despite recent advancements in preclinical and clinical studies, there is currently no effective therapy that can reverse muscle loss.

**Figure 5:**
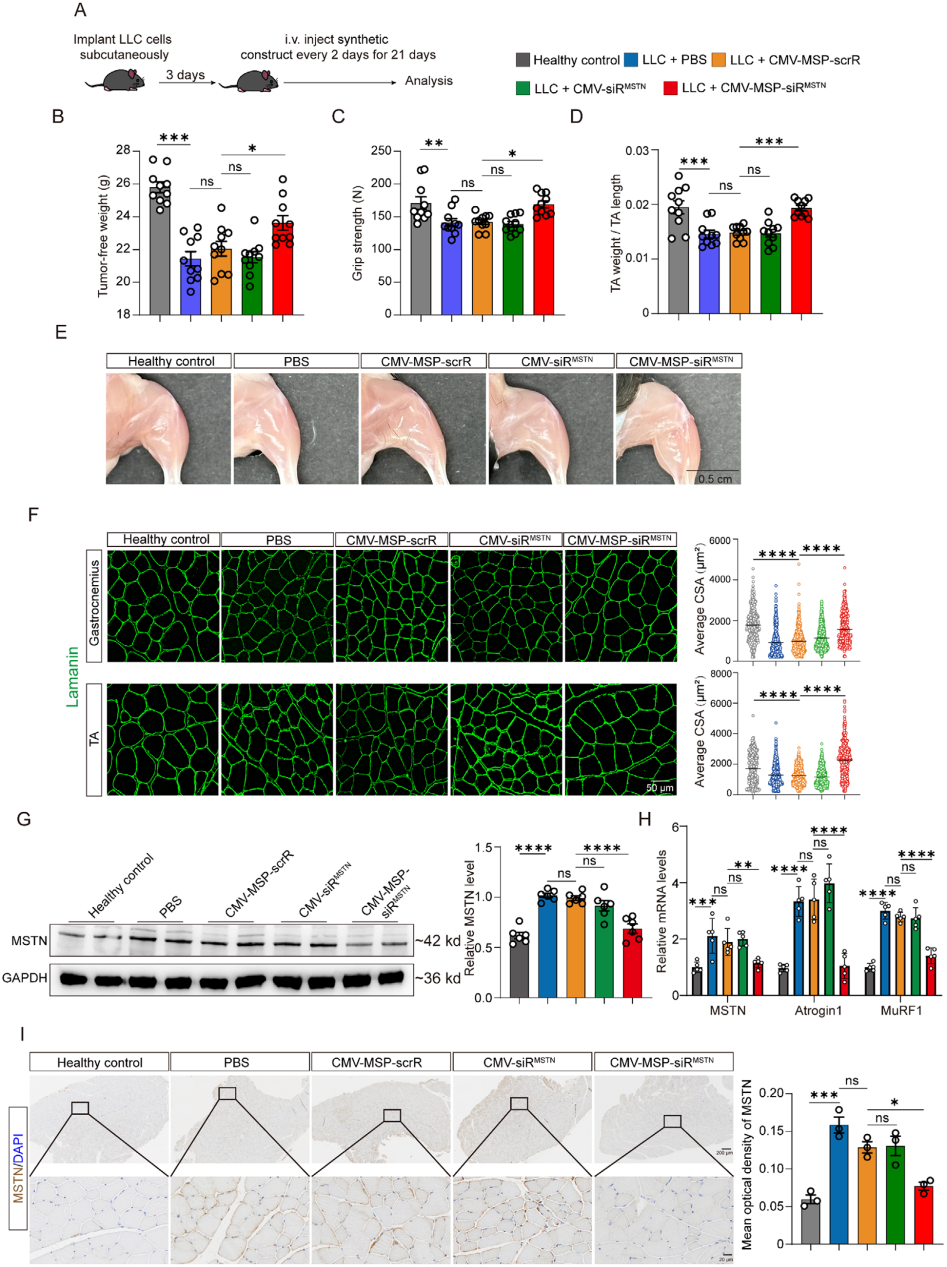
Restoration of Muscle Mass and Muscle Function in Lewis lung carcinoma (LLC) Tumor‐Bearing Mice by In Vivo Self‐Assembled MSTN‐siRNA. A) Schematic of the experimental design. A cancer‐induced muscle atrophy model was generated by subcutaneously inoculating 10^6^ LLC cells. Three days later, mice were administered synthetic constructs every 2 days for 21 days to evaluate their effectiveness in alleviating cancer‐induced muscle atrophy. B) Body weight changes (n = 10). C) Muscle function was assessed using grip strength measurement (n = 10). D) Determination of TA muscle weight (n = 10). E) Representative images of hindlimbs from each group. Scale bar = 1 cm. F) Laminin immunofluorescent staining of cross‐sections of gastrocnemius and TA muscles (left panel) and corresponding cross‐sectional area statistics. The scatter plot analyzes over 500 muscle fibers from 5 mice, with average values shown. Scale bar = 50 µm. G) Western blot analysis of MSTN protein levels in TA muscles. Representative Western blot (left panel) and densitometric analysis (right panel) are shown (n = 6). H) Quantitative RT‐PCR analysis of MSTN, Atrogin1, and MuRF1 mRNA levels in TA muscles (n = 5). I) Representative images of IHC staining for MSTN in TA muscle sections and corresponding quantification (n = 3). Scale bar = 200 µm (upper panel) and 20 µm (lower panel). Data are presented as mean ± SEM. Significance was determined using one‐way ANOVA followed by Bonferroni's multiple comparisons test for panels B–D and F–I. ^*^ *p* < 0.05; ^**^ *p* < 0.01; ^***^ *p* < 0.001; ^****^ *p* < 0.0001; ns = not significant.

Previous studies have investigated the phenomenon and molecular mechanisms of muscle wasting in cancer cachexia, and have shown that the activation of the MSTN signaling pathway is a notable contributor to muscle atrophy induced by cancer.^[^ ^23^ ^]^ To investigate the anti‐muscle wasting effect of the CMV‐MSP‐siR^MSTN^ construct in Lewis Lung Cancer (LLC) tumor‐bearing mice, we established a mouse model by subcutaneously inoculating LLC cells.^[^ ^24^ ^]^ The synthetic constructs were administered to mice three days post‐subcutaneous injection of ≈10^6^ LLC cells in the right flank, with subsequent injections every other day for a three‐week period (**Figure** **5**A). The results showed that the LLC cancer cell‐induced muscle atrophy was alleviated by injection of CMV‐MSP‐siR^MSTN^ construct, as reflected by a significant increase in tumor‐free body weight, grip strength, and TA weight in tumor‐bearing mice (Figure 5B–D). The atrophy of hindlimb musculature in tumor‐bearing mice was significantly mitigated in the group treated with the CMV‐MSP‐siR^MSTN^ construct, which demonstrates a clear contrast to other counterparts (Figure 5E). Laminin staining of gastrocnemius and TA muscle showed a significant restoration of muscle fiber CSA in the mice treated with CMV‐MSP‐siR^MSTN^ construct, to a level that is nearly comparable to the healthy control (Figure 5F). Moreover, molecular level of MSTN and its downstream effectors were reduced noticeably, reflecting in protein level of MSTN and mRNA levels of MSTN, MuRF1 and Atrogin1 (Figure 5G,H). Additionally, immunohistochemistry staining of MSTN in the TA muscle suggested a significant reduction in MSTN protein level in the mice treated with the CMV‐MSP‐siR^MSTN^ construct, and the level was similar to that of the healthy control group (Figure 5I). Overall, the IVSA‐siRNA targeting MSTN demonstrates a potential therapeutic effect in mitigating muscle atrophy in tumor‐bearing mice, suggesting a beneficial intervention for this condition.

### 2.7 Evaluation of the Therapeutic Effects of IVSA‐siRNA Targeting MSTN in Dex‐Induced Muscle Atrophy Model

Corticosteroids are extensively utilized in anti‐inflammatory therapies.^[^ ^25^ ^]^ Dex is a potent synthetic glucocorticoid widely used in the treatment of various inflammatory and immune‐mediated disorders.^[^ ^26^ ^]^ However, chronic administration of high doses of Dex is associated with significant muscle atrophy. This Dex‐induced muscle atrophy is characterized by a dose‐dependent upregulation of MSTN expression.^[^ ^27^ ^]^ Importantly, the deletion of MSTN has been shown to prevent the muscle atrophy induced by Dex, highlighting MSTN as a potential therapeutic target for preventing Dex‐induced atrophy.^[^ ^28^ ^]^

**Figure 6:**
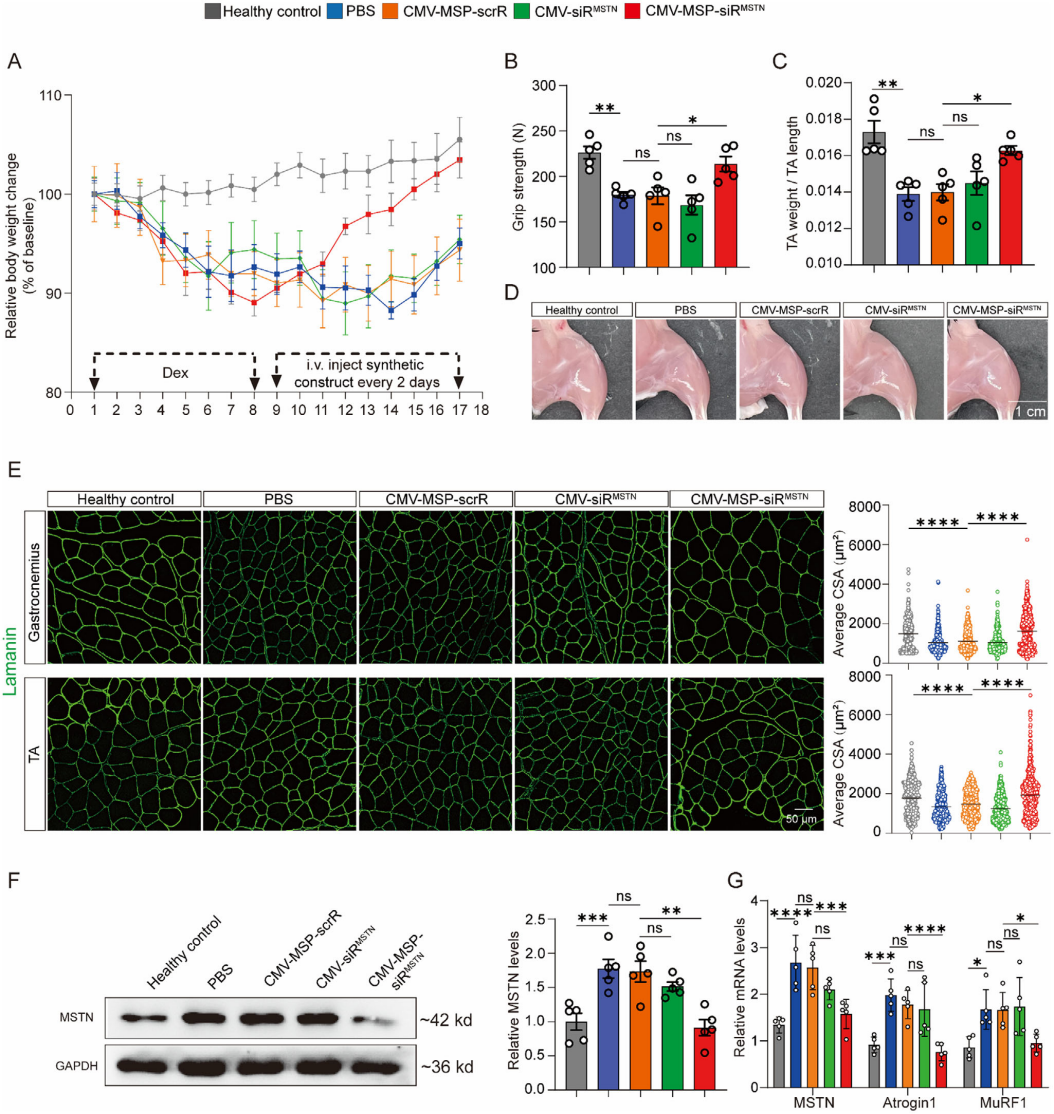
Restoration of Muscle Mass and Muscle Function in Dex‐Induced Muscle Atrophy Mice by In Vivo Self‐Assembled MSTN‐siRNA. A Dex‐induced muscle atrophy model was generated by administering high‐dose Dex (25 mg kg^−1^) via intraperitoneal injection for 7 consecutive days. Subsequently, mice were treated with synthetic constructs 5 times over 9 days to evaluate their effectiveness in alleviating Dex‐induced muscle atrophy. A) Body weight curve with schematic of the experimental design (n = 5). B) Muscle function assessed using grip strength measurement (n = 5). C) Determination of TA muscle weight (n = 5). D) Representative images of hindlimbs from each group. Scale bar = 1 cm. E) Laminin immunofluorescent staining of cross‐sections of gastrocnemius and TA muscles (left panel) and corresponding cross‐sectional area statistics. The scatter plot analyzes over 500 muscle fibers from 5 mice, with average values shown. Scale bar = 50 µm. F) Western blot analysis of MSTN protein levels in TA muscles. Representative Western blot (left panel) and densitometric analysis (right panel) are shown (n = 6). G) Quantitative RT‐PCR analysis of MSTN, Atrogin1, and MuRF1 mRNA levels in TA muscles (n = 5). Data are presented as mean ± SEM. Significance was determined using one‐way ANOVA followed by Bonferroni's multiple comparisons test for panels B, C, E, F, and G. ^*^ *p* < 0.05; ^**^ *p* < 0.01; ^***^ *p* < 0.001; ^****^ *p* < 0.0001; ns = not significant.

To investigate the therapeutic role of the CMV‐MSP‐siR^MSTN^ construct in Dex‐induced muscle atrophy, To establish a Dex‐induced muscle atrophy model, C57BL/6J male mice were intraperitoneally injected with Dex once daily for 7 consecutive days. Beginning on day 8, synthetic constructs were intravenously administered every other day, for a total of six doses. As expected, Dex treatment led to a significant reduction in body weight compared to the healthy control (≈10%). However, treatment with the CMV‐MSP‐siR^MSTN^ construct mitigated this weight loss in Dex‐administered mice (**Figure** **6**A). Grip strength and TA muscle weight were significantly improved in CMV‐MSP‐siR^MSTN^ construct‐treated group compared to other groups (Figure 6B,C). Alleviated skeletal muscle atrophy was also evident in the increased size of hindlimb muscles in the mice treated with CMV‐MSP‐siR^MSTN^ construct (Figure 6D). Immunofluorescence staining for Laminin revealed that Dex administration significantly reduced the CSA of gastrocnemius and TA muscles, whereas injection of the CMV‐MSP‐siR^MSTN^ construct alleviated Dex‐induced atrophy, as indicated by significantly larger CSA compared to other groups (Figure 6E). Additionally, Western blot analysis showed a significant increase in MSTN levels in Dex‐treated groups compared to healthy controls, while the injection of CMV‐MSP‐siR^MSTN^ construct significantly reduced MSTN levels in TA muscles (Figure 6F). Consistent with this, MSTN mRNA levels and the expression of its downstream effectors, MuRF1 and Atrogin1, were markedly increased in Dex‐treated groups, whereas the injection of CMV‐MSP‐siR^MSTN^ construct significantly reduced them (Figure 6G). Taken together, these findings demonstrate that the CMV‐MSP‐siR^MSTN^ construct effectively alleviates Dex‐induced muscle atrophy.

### 2.8 Evaluation of the Toxicity and Safety of IVSA‐siRNA Targeting MSTN

To assess the toxicity and safety of the CMV‐MSP‐siR^MSTN^ construct, we separately administered PBS, CMV‐MSP‐scrR, CMV‐siR^MSTN^, and CMV‐MSP‐siR^MSTN^ to four groups of mice, with injections given every two days for a total of seven times. After the treatment, peripheral blood and multiple organs were collected from the sacrificed mice. Biochemical markers for liver functions, including alanine transaminase (ALT), aspartate aminotransferase (AST), albumin (ALB), and total bilirubin (TBIL), as well as indicators for cardiac and kidney injuries, such as lactate dehydrogenase (LDH) and serum creatinine (CREA) and blood urea nitrogen (BUN), showed no significant differences among all groups (Figure S6A, Supporting Information). Similarly, peripheral blood counts, including red blood cells (RBC), white blood cells (WBC), and platelets (PLT), remained consistent across different groups (Figure S6B, Supporting Information). Histological examination of the liver, lung, kidney, and spleen confirmed the non‐toxicity and safety of the synthetic constructs, as no noticeable tissue damage was observed in these organs (Figure S6C, Supporting Information). In conclusion, these results suggest that the IVSA‐siRNA targeting MSTN functions in a non‐toxic and biocompatible manner in vivo.

## 3 Discussion

In this study, we developed a synthetic biology‐based strategy that leverages the host's endogenous miRNA assembly and sEV transport machinery to achieve efficient siRNA delivery to skeletal muscle. Tail vein injection of the synthetic construct led to in vivo self‐assembly of MSTN siRNA‐loaded sEVs in the liver. These sEVs, with the muscle‐targeting MSP peptide displayed on the outer membrane, were naturally secreted into the circulation and selectively accumulated in skeletal muscle, enabling targeted delivery of MSTN siRNA.

Compared to traditional strategies using lipid nanoparticles (LNPs) or cell culture‐derived sEVs for siRNA delivery, our approach offers a major advantage: the ability to achieve broad and efficient gene silencing across multiple skeletal muscles through systemic administration. In contrast, existing LNP‐ or cell culture‐derived sEV‐based delivery methods typically require local intramuscular injections, limiting their effects to specific muscles and thus reducing their clinical applicability. The systemic delivery efficiency and multi‐muscle targeting capability of our platform were validated in GFP transgenic mice, where tail vein injection of GFP siRNA resulted in significant GFP knockdown in multiple muscles, including the quadriceps, gastrocnemius, and tibialis anterior.

In addition to improved delivery efficiency and tissue accessibility, the IVSA system further addresses challenges related to production scalability and safety. IVSA uses tail vein injection of a naked plasmid encoding the synthetic construct, allowing the host to autonomously generate therapeutic sEVs in vivo without the need for exogenous vesicle preparation. This eliminates the labor‐intensive steps of sEV isolation and purification, and avoids compositional variability often encountered with cell culture‐derived sEVs. Moreover, as the sEVs are endogenously produced by the host itself, the risk of immunogenicity is minimized. This advantage is consistent with our observation that repeated administration of the synthetic constructs was well tolerated in vivo, with no evidence of toxicity, and maintained high biocompatibility.

Although our study demonstrates that IVSA‐derived sEVs carrying MSTN‐siRNA effectively alleviate muscle atrophy in mouse models, several limitations should be noted. First, the cellular uptake and intracellular trafficking mechanisms of MSP‐tagged sEVs remain unclear. Our data indicate that MSP significantly enhances skeletal muscle delivery; however, whether MSP mediates receptor‐dependent endocytosis or direct membrane fusion has not been determined. Moreover, the precise mechanism by which MSP‐sEVs avoid lysosomal degradation remains elusive. Future studies will be required to elucidate these pathways, as understanding these mechanisms is essential for optimizing IVSA‐mediated siRNA delivery. Second, the systemic biodistribution of IVSA‐derived sEVs warrants consideration. Using the IVSA system to express GFP siRNA, we observed significant knockdown of GFP protein not only in skeletal muscle but also in other organs, including the liver, lung, and kidney. Nevertheless, since MSTN expression is predominantly restricted to skeletal muscle and cardiac muscle, and MSP primarily enhances skeletal muscle uptake without affecting cardiac delivery, the functional knockdown effect of MSTN‐siRNAs is expected to remain largely confined to skeletal muscle where MSTN is highly expressed. Third, the dose–response relationship of IVSA‐siRNA therapy has not been fully characterized. Future studies employing multiple dosing regimens in animal models are needed to determine whether higher doses can further enhance therapeutic efficacy without compromising safety. Finally, preclinical validation in larger animal models will be critical before clinical translation. Although MSTN is highly conserved between mice and humans, additional studies in non‐human primates are required to comprehensively evaluate pharmacokinetics, biodistribution, therapeutic efficacy, and long‐term safety.

Taken together, this study introduces a groundbreaking method for delivering IVSA‐siRNA specifically to skeletal muscle. By incorporating a muscle‐targeting peptide tag and MSTN‐siRNA expression cassette, this approach effectively targets and delivers MSTN‐siRNA to skeletal muscle tissues via MSTN‐siRNA‐encapsulating sEVs. The resulting degradation of MSTN mRNA and reduction in MSTN protein demonstrated efficacy in alleviating muscle wasting in models of cancer‐ and dex‐induced muscle atrophy. These results underscore the potential of this targeted delivery system to address muscle degeneration and highlight opportunities for future advancements. Further development of this system could enhance the precision and effectiveness of siRNA delivery, paving the way for novel therapeutic interventions in muscle‐related diseases.

## 4 Experimental Model and Subject Details

### Animals

C57BL/6J and GFP‐transgenic mice were obtained from GemPharmatech Co., Ltd. (Nanjing, China). The animals were maintained on a 12‐h light/dark cycle (lights on at 7:00 a.m.) with unrestricted access to food and water. Post‐treatment, mice were euthanized for the collection of tissues and peripheral blood samples. All experimental procedures were conducted in accordance with the guidelines set by the Institutional Animal Care and Use Committee (IACUC) of Nanjing University (Approval No. IACUC‐2410002‐2).

### Summary of Animal Numbers Used in Each Study

In the study Characterization of self‐assembled MSTN‐siRNA in an ex vivo model, a total of 75 C57BL/6J mice were used. Among them, 12 mice were used to quantify absolute siRNA levels in the liver (Figure 2B), 24 mice were used to assess siRNA levels in sEVs at various time points after intravenous injection (Figure 2C,D), 15 mice were used to validate sEV encapsulation of siRNA (Figure 2E), and the remaining 24 mice were used to isolate serum sEVs for co‐culture experiments with C2C12 myotubes (Figure 2E–G). In the study Tracking and visualization of the delivery of self‐assembled MSTN‐siRNA to skeletal muscle, a total of 39 C57BL/6J mice and 24 GFP‐transgenic mice were used. Among these, 27 mice (18 donors and 9 recipients) were used to track PKH26‐labeled serum sEVs in the gastrocnemius muscle (Figure 3B). An additional 12 mice were used for in situ hybridization of siRNA in the tibialis anterior muscle (Figure 3C). Furthermore, 12 GFP‐transgenic mice (3 per group) were used to detect GFP fluorescence across different tissues (Figure 3D, Figure S4, Supporting Information), and another 12 GFP‐transgenic mice (3 per group) were used to evaluate siRNA‐LNP knockdown efficiency in the tibialis anterior muscle (Figure S5, Supporting Information). In the study Modulation of skeletal muscle mass in healthy wild‐type mice by in vivo self‐assembled MSTN‐siRNA, a total of 28 C57BL/6J mice were used (7 per group, Figure 4). In the study Restoration of muscle mass and function in Lewis lung carcinoma (LLC) tumor‐bearing mice by in vivo self‐assembled MSTN‐siRNA, a total of 50 C57BL/6J mice were used (10 per group, Figure 5). In the study Restoration of muscle mass and muscle function in Dex‐induced muscle atrophy mice by in vivo self‐assembled MSTN‐siRNA, a total of 25 C57BL/6J mice were used (Figure 6). Additionally, the number of animals used in each experiment has been explicitly stated in the corresponding figure legends using “n” to ensure clarity and reproducibility.

### Methods Details—Design and Construction of the Synthetic Constructs Targeting MSTN

The CMV‐siR^MSTN^ construct was engineered by integrating an MSTN‐targeting siRNA sequence into a 166‐bp pre‐miR‐155 backbone, which was structurally modified to preserve base pairing integrity. Details of the inserted sequences are provided in Table S1 (Supporting Information). The CMV‐MSP‐siR^MSTN^ construct was designed to express a muscle‐specific peptide (MSP) fused to the N‐terminus of lysosome‐associated membrane glycoprotein 2b (Lamp2b). The MSP‐Lamp2b fusion and the MSTN‐siRNA embedded within the pre‐miR‐155 backbone were both placed under the control of the CMV promoter. A scrambled RNA construct served as the negative control. All constructs (DNA plasmids) were synthesized by GenScript (Piscataway, NJ, USA) and dissolved in 1x PBS. The scaffold and plasmid map are depicted in Figure S7 (Supporting Information). Plasmids were transformed into *Escherichia coli* DH5α competent cells and cultured overnight in LB medium at 37 °C with shaking. Following incubation, plasmids were extracted and purified using the EndoFree Plasmid Kit V2 (Tiangen, DP120), following the manufacturer's protocol. Sequencing was subsequently performed to verify the correct insertion of the recombinant gene cassette.

### Cell Culture

The human embryonic kidney cell line HEK293T and the murine myoblast cell line C2C12 were obtained from the Shanghai Institute of Cell Biology, Chinese Academy of Sciences. Cells were cultured at 37 °C in a humidified atmosphere containing 5% CO_2_ in high‐glucose Dulbecco's modified Eagle medium (DMEM; 4.5 g L^−1^ glucose, Gibco), supplemented with 10% fetal bovine serum (FBS; Gibco), 100 U mL^−1^ penicillin, and 100 mg mL^−1^ streptomycin. For the induction of C2C12 myoblast differentiation into myotubes, cells were allowed to reach ≈70% confluence before the culture medium was replaced with differentiation medium containing 2% horse serum. The medium was refreshed every two days until the formation of myotubes was observed.

### In Vitro Transfection of the Synthetic Constructs

The synthetic constructs were transfected into HEK293T or C2C12 myotube cells using Lipofectamine 2000 (Invitrogen) following the manufacturer's protocol. Total RNA and protein were extracted from the cells 48 h post‐transfection. Extracts were then stored at −80 °C for subsequent analysis.

### In Vivo Injection of Synthetic Constructs

The synthetic constructs, as naked DNA plasmids, were administered to mice via tail vein injection at a dosage of 5 mg kg^−1^. The injection was performed with a volume of 100–200 µL and was completed in ≈3 s.

### Isolation and Characterization of Serum sEVs

Mice were anesthetized using an intraperitoneal injection of 1.25% Avertin. Following the onset of full anesthesia, blood was drawn from the retro‐orbital venous plexus and collected into 1.5 mL microcentrifuge tubes. The blood samples were allowed to clot at room temperature for 1 h. Subsequently, samples were centrifuged at 3000 × g at 4 °C for 15 min to separate the supernatant. To remove large EVs, the serum was further centrifuged at 10 000 × g at 4 °C for 1 h. The resulting supernatant was diluted with PBS at a 1:10 ratio and filtered through a 0.22 µm filter. The filtrate was then subjected to high‐speed centrifugation at 1 20 000 × g at 4 °C for 2 h to isolate the sEV pellet.

Several techniques were employed to characterize sEVs, as described below. Nanoparticle Tracking Analysis (NTA) using the NanoSight NS300 system (NanoSight Technology) was utilized to assess the size distribution of sEVs, revealing a predominant peak ≈110 nm in diameter. Transmission Electron Microscopy (TEM) with the HT7700 system (Japan) provided detailed morphological insights, confirming the presence of typical cup‐shaped structures ranging from 30 to 150 nm. Western blot analysis was performed using antibodies against Alix (Santa Cruz, sc‐166952), CD9 (Santa Cruz, sc‐20048), and CD63 (Santa Cruz, sc‐5275) to evaluate the enrichment of sEVs.

### Incubation of Serum sEVs with C2C12 Myotubes

C2C12 myoblasts were differentiated into myotubes over 4 days using differentiation medium. The differentiated myotubes were then co‐cultured with 30 µg of serum‐derived sEVs for 48 h. Following the co‐culture period, the myotubes were harvested for subsequent analysis of MSTN expression and for measurement of myotube diameter.

### In Vivo Tracking of sEVs

To label sEVs, sEVs (40 µg total protein) were incubated with 2.5 µL of PKH26 red fluorescent dye (Sigma–Aldrich) according to the manufacturer's instructions. The labeled sEVs were then injected via tail vein for in vivo tracking. After 16 h, skeletal muscle tissues from the recipient mice were collected, frozen, sectioned, and examined to assess the distribution of the labeled sEVs.

### In Situ Hybridization of siRNA

Paraffin‐embedded sections of the mice tibialis anterior were deparaffinized by incubating in xylene for 5 min, twice, followed by two 2‐min incubations in ethanol. The sections were then dried in an oven at 60 °C for 5 min. Post‐fixation was performed using 10% formaldehyde. The sections were treated with RNAscope Hydrogen Peroxide solution for 10 min, followed by a 15‐min incubation in RNAscope target retrieval buffer heated to 99 °C. After rinsing with distilled water, the sections were treated with 100% ethanol for 3 min at room temperature. Subsequent treatment with RNAscope Protease Plus was carried out, followed by a 30‐min incubation at 40 °C in a HybEZ oven (ACD Biotech). After washing with distilled water, the specific probe (ACD Biotech) was applied to the slides and incubated at 40 °C for 2 h. Detection of target probes was performed using the miRNAscope HD Reagent Kit‐RED, with sequential incubations with Amp1‐5. Finally, the slides were stained with 50% hematoxylin for nuclei visualization and scanned with an Olympus VS200 for further analysis.

### GFP Fluorescence Intensity Detection

GFP‐transgenic mice were randomly assigned to different groups and received intravenous injections of PBS, CMV‐MSP‐scrR, CMV‐siR^GFP^, or CMV‐MSP‐siR^GFP^, administered seven times. Tissues were harvested 24 h after the final injection, fixed, and embedded in OCT for frozen sectioning to assess GFP fluorescence.

### Grip Strength Test

The grip strength test was conducted following established protocols.^[^ ^29^ ^]^ In brief, the mouse was held by the tail, allowing its forelimbs to grasp the grid of a meter. The mouse was then slowly pulled backward until it released its grip on the grid, with the peak force recorded. Each mouse was subjected to three to five trials, with brief rest periods between trials. The average grip strength was calculated from these measurements for subsequent analysis.

### Quantitative Reverse Transcription PCR (Quantitative RT‐PCR)

Total RNA was isolated from mouse tissue or cultured cells using TRIzol (Invitrogen, Carlsbad, CA, USA) according to the manufacturer's instructions.

To detect mRNAs, 0.5 µg of total RNA was reverse transcribed to cDNA using HiScript III RT SuperMix (Vazyme, China) according to the manufacturer's instruction. Quantitative RT‐PCR was performed using ChamQ Universal SYBR qPCR Master Mix (Vazyme) on a LightCycler 480 II system (Roche, Mannheim, Germany) according to the manufacturer's instruction. The ΔΔCT method was used to analyze mRNA levels. GAPDH served as the internal control. All samples were biologically replicated 3 times. Primer sequences are listed in Table S2 (Supporting Information).

To detect siRNAs, 100 ng of total RNA was reverse transcribed to cDNA using miRNA 1st Strand cDNA Synthesis Kit (by stem‐loop) (Vazyme). Quantitative RT‐PCR was performed using miRNA Universal SYBR qPCR Master Mix (Vazyme) on a LightCycler 480 II system (Roche). Relative siRNA expression was normalized to U6 small nuclear RNA (snRNA). All samples were biologically replicated 3 times. Primer sequences are listed in Supplementary Table S2 (Supporting Information).

For quantification of the absolute levels of MSTN‐siRNA, TaqMan miRNA probes (Applied Biosystems) was applied according to the manufacturer's instructions. Briefly, 0.5 µg of total RNA was reverse‐transcribed into cDNA using a customized stem‐loop reverse transcription primer (Applied Biosystems) and avian myeloblastosis virus (AMV) reverse transcriptase (TaKaRa). Real‐time PCR was run with a TaqMan MicroRNA Assay (Applied Biosystems) and a LightCycler 480 II system. Synthetic single‐stranded MSTN‐siRNA was serially diluted to generate a standard curve using quantitative RT‐PCR. By referring to the standard curve, the concentrations of MSTN‐siRNA in sEVs isolated from serum were calculated and are presented as the absolute amounts of MSTN‐siRNA in per µg of sEVs (pmol µg^−1^ sEV). All reactions were run in triplicate. MSTN‐siRNA probe was listed in Supplementary Table S2 (Supporting Information).

### Western Blot

Protein extracts were separated by SDS‐PAGE (Yeasen) and subsequently transferred to a PVDF membrane. The membrane was blocked with 5% non‐fat milk in TBST for 1 h at room temperature. Following blocking, the membrane was incubated overnight at 4 °C with the primary antibody against MSTN (Affinity, #DF13273). After washing with TBST, the membrane was incubated with the appropriate secondary antibodies for 1 h at room temperature. Protein bands were visualized using an enhanced chemiluminescence (ECL) detection system (Fdbio Science) and imaged with a chemiluminescence imaging device. Band intensity was quantified using ImageJ software.

### Immunofluorescence

Skeletal muscle sections (8 µm thick) or cultured myotubes were fixed with 4% paraformaldehyde, permeabilized with 0.3% Triton X‐100, and blocked with 5% normal serum in PBS containing 1% BSA. Primary antibodies (Anti‐Laminin, Abcam, ab11575; Anti‐MyHC, R&D Systems, MAB4470) were incubated overnight at 4 °C. Following primary antibody incubation, the sections were treated with fluorescently labeled secondary antibodies for 1 h at room temperature. Skeletal muscle sections were counterstained with DAPI to visualize nuclei and mounted with anti‐fade mounting medium. Images were acquired using a fluorescence microscope.

### Immunohistochemistry (IHC)

Skeletal muscle sections (8 µm thick) embedded in paraffin were first deparaffinized by heating at 65 °C and then subjected to sequential dehydration using xylene and ethanol. Antigen retrieval was achieved by incubating the sections in citrate buffer (pH 6.0) at 95 °C for 20 min. After cooling and washing with PBS, endogenous peroxidase activity was inhibited using a 3% hydrogen peroxide solution at room temperature. To prevent nonspecific binding, the sections were blocked with 1% BSA in PBS and then incubated overnight at 4 °C with a primary anti‐myostatin antibody (Affinity, #DF13273) diluted in the blocking buffer. On the following day, after washing, sections were exposed to a biotinylated secondary antibody for 1 h at room temperature. Subsequent to additional washes, staining was completed using streptavidin‐conjugated HRP and DAB substrate. Finally, sections were counterstained with hematoxylin, dehydrated, mounted with coverslips, and visualized using a light microscope.

### Biocompatibility Evaluation of Synthetic Constructs

Male mice (7 weeks old) were acclimated for one week before receiving tail vein injections of PBS or 5 mg k^−1^g synthetic constructs every other day for two weeks. After the final injection, mice were anesthetized, and peripheral blood and tissues were collected 24 h later. Serum was analyzed for ALT, AST, ALP, LDH, TBIL, CREA, and BUN using a Chemray 800 analyzer. Blood cell counts for RBCs, WBCs, and PLTs were recorded. Fixed tissues were paraffin‐embedded, sectioned, and stained with H&E to evaluate tissue damage.

### Statistical Analysis

All statistical analyses were performed using GraphPad Prism 8 software. All data are presented as the mean ± standard error of the mean (SEM) from at least three independent experiments. Multiple group comparisons were analyzed by using one‐way ANOVA followed by Bonferroni's multiple comparisons test. Significance was accepted as follows: ^*^ *P* < 0.05; ^**^ *P* < 0.01; ^***^ *P* < 0.001, and ^****^ *P* < 0.0001. Details on the statistics are indicated in the figure legends.

## Conflict of Interest

The authors declare no conflict of interest.

## Author Contributions

X.C., X.Y., and L.S. designed the study. X.Y., A.A., J.C., Q.,S. L.Y., J.T., Y.C., L.Z., and J.G. performed experiments. X.C., X.Y., A.A., and J.C. analyzed the data. J.M., Z.L., F.Y., and C.Z. designed experiments and edited the manuscript. X.C. and X.Y. designed experiments and wrote the manuscript.

## Supporting information



Supporting Information

## Data Availability

The data that support the findings of this study are available from the corresponding author upon reasonable request.

## References

[1] Furrer, R. , C. Handschin , Annu. Rev. Pharmacol. Toxicol. 2019, 59, 315. 30148697 10.1146/annurev-pharmtox-010818-021041 PMC6701981

[2] S. K. Powers , G. S. Lynch , K. T. Murphy , M. B. Reid , I. Zijdewind , Med. Sci. Sports. Exerc. 2016, 48, 2307. 27128663 10.1249/MSS.0000000000000975 PMC5069191

[3] A. C. McPherron , A. M. Lawler , S. J. Lee , Nature 1997, 387, 83. 9139826 10.1038/387083a0

[4] S. J. Lee , J. Clin. Invest. 2021, 131, 148372. 33938454 10.1172/JCI148372 PMC8087205

[5] Y. S. Gallot , A.‐C. Durieux , J. Castells , M. M. Desgeorges , B. Vernus , L. Plantureux , D. Rémond , V. E. Jahnke , E. Lefai , D. Dardevet , G. Nemoz , L. Schaeffer , A. Bonnieu , D. G. Freyssenet , Cancer. Res. 2014, 74, 7344. 25336187 10.1158/0008-5472.CAN-14-0057

[6] D. Verzola , C. Barisione , D. Picciotto , G. Garibotto , L. Koppe , Kidney. Int. 2019, 95, 506. 30598193 10.1016/j.kint.2018.10.010

[7] Y. Hong , J. H. Lee , K. W. Jeong , C. S. Choi , H.‐S. Jun , J Cachexia. Sarcopenia. Muscle. 2019, 10, 903. 31020810 10.1002/jcsm.12434 PMC6711418

[8] S. M. Hoy , Drugs 2018, 78, 1625. 30251172 10.1007/s40265-018-0983-6

[9] B. Peng , Y. Yang , Z. Wu , R. Tan , T. T. Pham , E. Y. M. Yeo , M. Pirisinu , M. K. Jayasinghe , T. C. Pham , K. Liang , N. Shyh‐Chang , M. T. N. Le , Mol. Ther. 2023, 31, 1418. 37016578 10.1016/j.ymthe.2023.03.036 PMC10188904

[10] J. G. van den Boorn , M. Schlee , C. Coch , G. Hartmann , Nat. Biotechnol. 2011, 29, 325. 21478846 10.1038/nbt.1830

[11] Z. Fu , X. Zhang , X. Zhou , U. Ur‐Rehman , M. Yu , H. Liang , H. Guo , X. Guo , Y. Kong , Y. Su , Y. Ye , X. Hu , W. Cheng , J. Wu , Y. Wang , Y. Gu , S.‐F. Lu , D. Wu , K. Zen , J. Li , C. Yan , C.‐Y. Zhang , X. Chen , Cell. Res. 2021, 31, 631. 33782530 10.1038/s41422-021-00491-z PMC8169669

[12] L. Zhang , T. Wu , Y. Shan , G. Li , X. Ni , X. Chen , X. Hu , L. Lin , Y. Li , Y. Guan , J. Gao , D. Chen , Y. Zhang , Z. Pei , X. Chen , Brain 2021, 144, 3421. 34918046 10.1093/brain/awab354 PMC8677541

[13] L. Yu , G. Fan , Q. Wang , Y. Zhu , H. Zhu , J. Chang , Z. Wang , S. Zhan , X. Hua , D. She , J. Huang , Y. Wang , J. Zhao , C.‐Y. Zhang , X. Chen , G. Zhou , Cell. Death. Dis. 2023, 14, 626. 37739958 10.1038/s41419-023-06159-3 PMC10516902

[14] Y. Sun , Y. Zhao , X. Ni , Y. Yang , Z. Fu , R. Liu , C.‐Y. Zhang , X. Chen , J. Control. Release. 2023, 358, 142. 37068521 10.1016/j.jconrel.2023.04.026

[15] T. I. Samoylova , B. F. Smith , Muscle. Nerve. 1999, 22, 460. 10204780 10.1002/(sici)1097-4598(199904)22:4<460::aid-mus6>3.0.co;2-l

[16] H. Yin , H. M. Moulton , C. Betts , Y. Seow , J. Boutilier , P. L. Iverson , M. J. A. Wood , Hum. Mol. Genet. 2009, 18, 4405. 19692354 10.1093/hmg/ddp395

[17] H. Yin , H. M. Moulton , C. Betts , T. Merritt , Y. Seow , S. Ashraf , Q. Wang , J. Boutilier , M. J. Wood , Mol. Ther. 2010, 18, 1822. 20700113 10.1038/mt.2010.151 PMC2951563

[18] C.‐Y. Yu , Z. Yuan , Z. Cao , B. Wang , C. Qiao , J. Li , X. Xiao , Gene. Ther. 2009, 16, 953. 19474807 10.1038/gt.2009.59 PMC2726895

[19] L. Alvarez‐Erviti , Y. Seow , H. Yin , C. Betts , S. Lakhal , M. J. A. Wood , Nat. Biotechnol. 2011, 29, 341. 21423189 10.1038/nbt.1807

[20] S. C. Bodine , E. Latres , S. Baumhueter , V. K.‐M. Lai , L. Nunez , B. A. Clarke , W. T. Poueymirou , F. J. Panaro , E. Na , K. Dharmarajan , Z.‐Q. Pan , D. M. Valenzuela , T. M. DeChiara , T. N. Stitt , G. D. Yancopoulos , D. J. Glass , Science 2001, 294, 1704. 11679633 10.1126/science.1065874

[21] S. Cohen , J. A. Nathan , A. L. Goldberg , Nat. Rev. Drug. Discov. 2015, 14, 58. 25549588 10.1038/nrd4467

[22] M. Ferrer , T. G. Anthony , J. S. Ayres , G. Biffi , J. C. Brown , B. J. Caan , E. M. Cespedes Feliciano , A. P. Coll , R. F. Dunne , M. D. Goncalves , J. Grethlein , S. B. Heymsfield , S. Hui , M. Jamal‐Hanjani , J. M. Lam , D. Y. Lewis , D. McCandlish , K. M. Mustian , S. O'Rahilly , N. Perrimon , E. P. White , T. Janowitz , Cell 2023, 186, 1824. 37116469 10.1016/j.cell.2023.03.028 PMC11059056

[23] T. Setiawan , I. N. Sari , Y. T. Wijaya , N. M. Julianto , J. A. Muhammad , H. Lee , J. H. Chae , H. Y. Kwon , J. Hematol. Oncol. 2023, 16, 54. 37217930 10.1186/s13045-023-01454-0 PMC10204324

[24] E. E. Talbert , M. C. Cuitiño , K. J. Ladner , P. V. Rajasekerea , M. Siebert , R. Shakya , G. W. Leone , M. C. Ostrowski , B. Paleo , N. Weisleder , P. J. Reiser , A. Webb , C. D. Timmers , D. S. Eiferman , D. C. Evans , M. E. Dillhoff , C. R. Schmidt , D. C. Guttridge , Cell. Rep. 2019, 28, P1612. 10.1016/j.celrep.2019.07.016 PMC6733019 31390573

[25] T. Rhen , J. A. Cidlowski , N. Engl. J. Med. 2005, 353, 1711. 16236742 10.1056/NEJMra050541

[26] A. Swierczek , W. J. Jusko , J. Pharmacol. Exp. Ther. 2023, 384, 455. 36631280 10.1124/jpet.122.001477 PMC9976795

[27] K. Ma , C. Mallidis , S. Bhasin , V. Mahabadi , J. Artaza , N. Gonzalez‐Cadavid , J. Arias , B. Salehian , Am. J. Physiol. Endocrinol. Metab. 2003, 285, E363. 12721153 10.1152/ajpendo.00487.2002

[28] H. Gilson , O. Schakman , L. Combaret , P. Lause , L. Grobet , D. Attaix , J. M. Ketelslegers , J. P. Thissen , Endocrinology 2007, 148, 452. 17038559 10.1210/en.2006-0539

[29] O. A. Meyer , H. A. Tilson , W. C. Byrd , M. T. Riley , Neurobehav. Toxicol. 1979, 1, 233. 551317

