# Supplementary material for: Silencing Myostatin Using In Vivo Self‐Assembled siRNA Protects Against Cancer‐ and Dexamethasone‐Induced Muscle Atrophy
Source: Adv Healthc Mater. 2025 Oct 3;15(4):e02186. doi: 10.1002/adhm.202502186 (PMC12836460; doi:10.1002/adhm.202502186)
Supplement: Supplementary file 1 — Supporting Information [file ADHM-15-0-s001.docx]

**Supplementary Materials for**

**Silencing myostatin using in vivo self-assembled siRNA protects against cancer- and dexamethasone-induced muscle atrophy**

**The file includes:**

Figure S1 to S7

Table S1 and S2

**
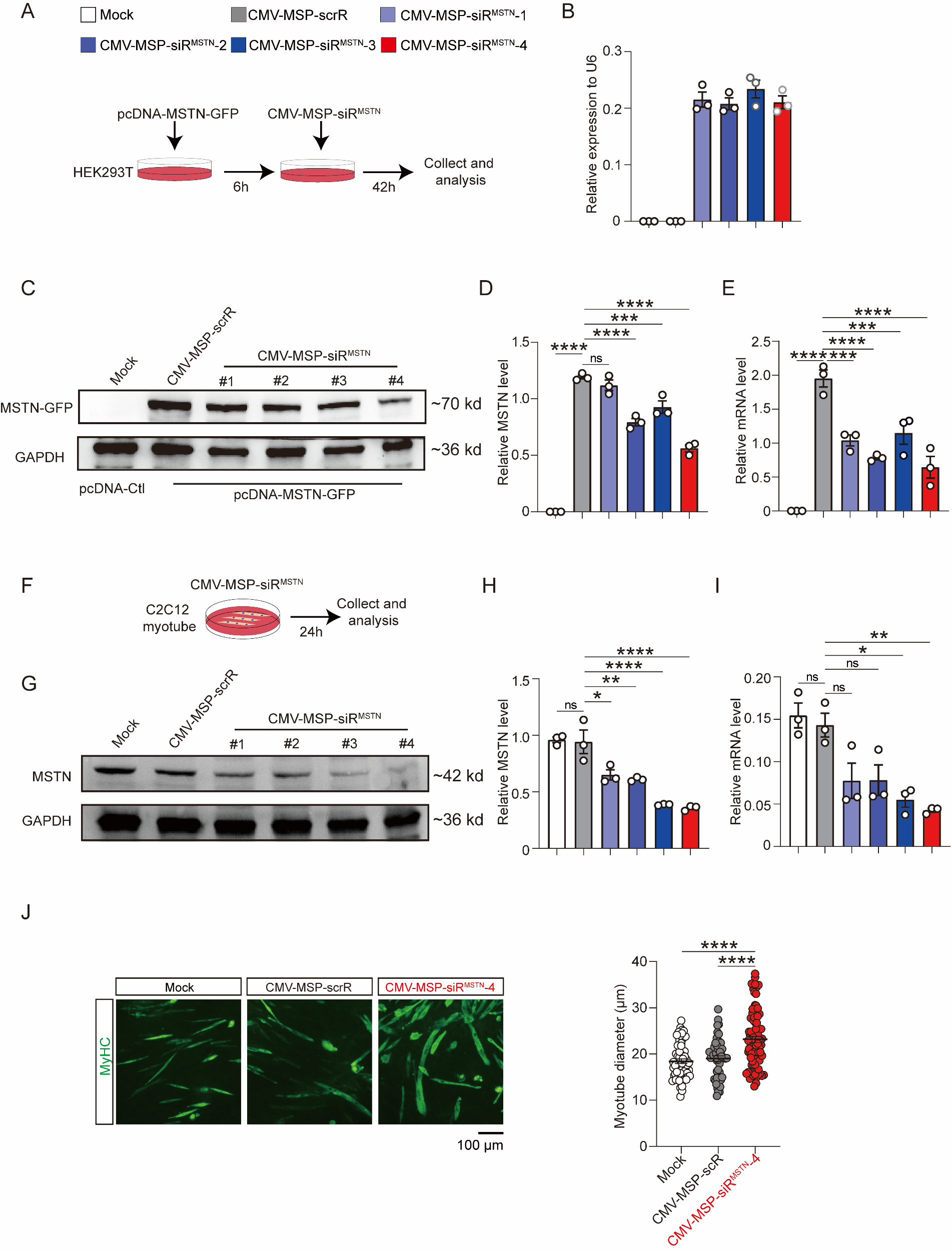
**

**Figure S1. Construction and characterization of the synthetic constructs.**

(A) Schematic of the experimental design. HEK293T cells were transfected with a control plasmid (pcDNA-Ctl) or a plasmid expressing GFP-tagged MSTN (pcDNA-MSTN-GFP). CMV-MSP-scrR, CMV-MSP-siR^MSTN^-1, -2, -3 or -4 constructs were transfected into cells already expressing pcDNA-MSTN-GFP 6 hours post-transfection. Cells were collected 42 hours after synthetic constructs transfection. (B) Quantitative RT-PCR analysis was performed to measure siRNA levels (n = 3). (C) Representative western blot image was shown to illustrate MSTN-GFP protein levels. (D) Quantitation of the MSTN-GFP protein levels (n = 3). (E) Quantitative RT-PCR analysis was performed to measure MSTN mRNA levels (n = 3). (F) Schematic of the experimental design. C2C12 myotubes were transfected with CMV-MSP-scrR, CMV-MSP-siR^MSTN^-1, -2, -3 or -4 constructs. Myotubes were collected 24 hours post-transfection. (G) Representative western blot image was shown to illustrate MSTN protein levels. (H) Quantitation of the MSTN protein levels (n = 3). (I) Quantitative RT-PCR analysis was performed to measure MSTN mRNA levels (n = 3). (J) Representative images of immunofluorescence for MyHC protein (Left panel) and corresponding diameter statistics (Right panel). The scatter plot analyzes more than 50 myotubes, with the average values shown in the figure. Scale bar: 100 μm. Values are presented as mean ± SEM. Significance was determined using one-way ANOVA followed by Bonferroni's multiple comparisons in C-J. *P < 0.05; **P < 0.01; ***P < 0.001; ****P < 0.0001; ns = not significant.


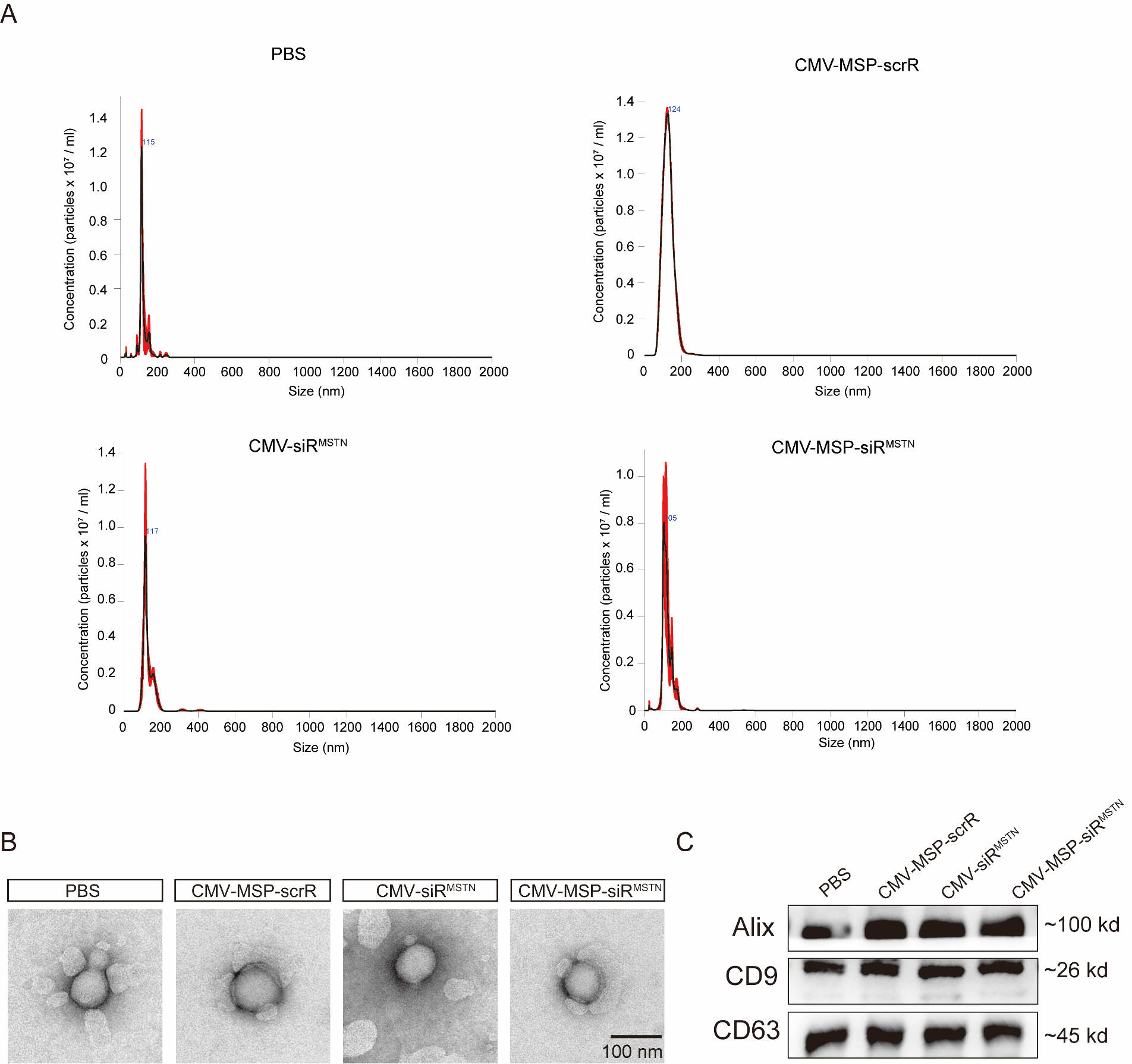


**Figure S2. Characterization of the properties of purified sEVs.**

C57BL/6J mice were intravenously injected with PBS or with 5 mg/kg CMV-scrR, CMV-MSP-siR^MSTN^ or CMV-MSP-siR^MSTN^ constructs every 2 days for a total of seven times, and then the sEVs were purified from mouse serum and characterized by using NTA and TEM. The enrichment of sEVs markers was analyzed by Western blot. (A) The size distribution and concentration of purified sEVs was determined by NTA. (B) Representative TEM images of purified sEVs. (C) Western blot analysis of specific sEVs markers (Alix, CD9 and CD63) in purified sEVs.


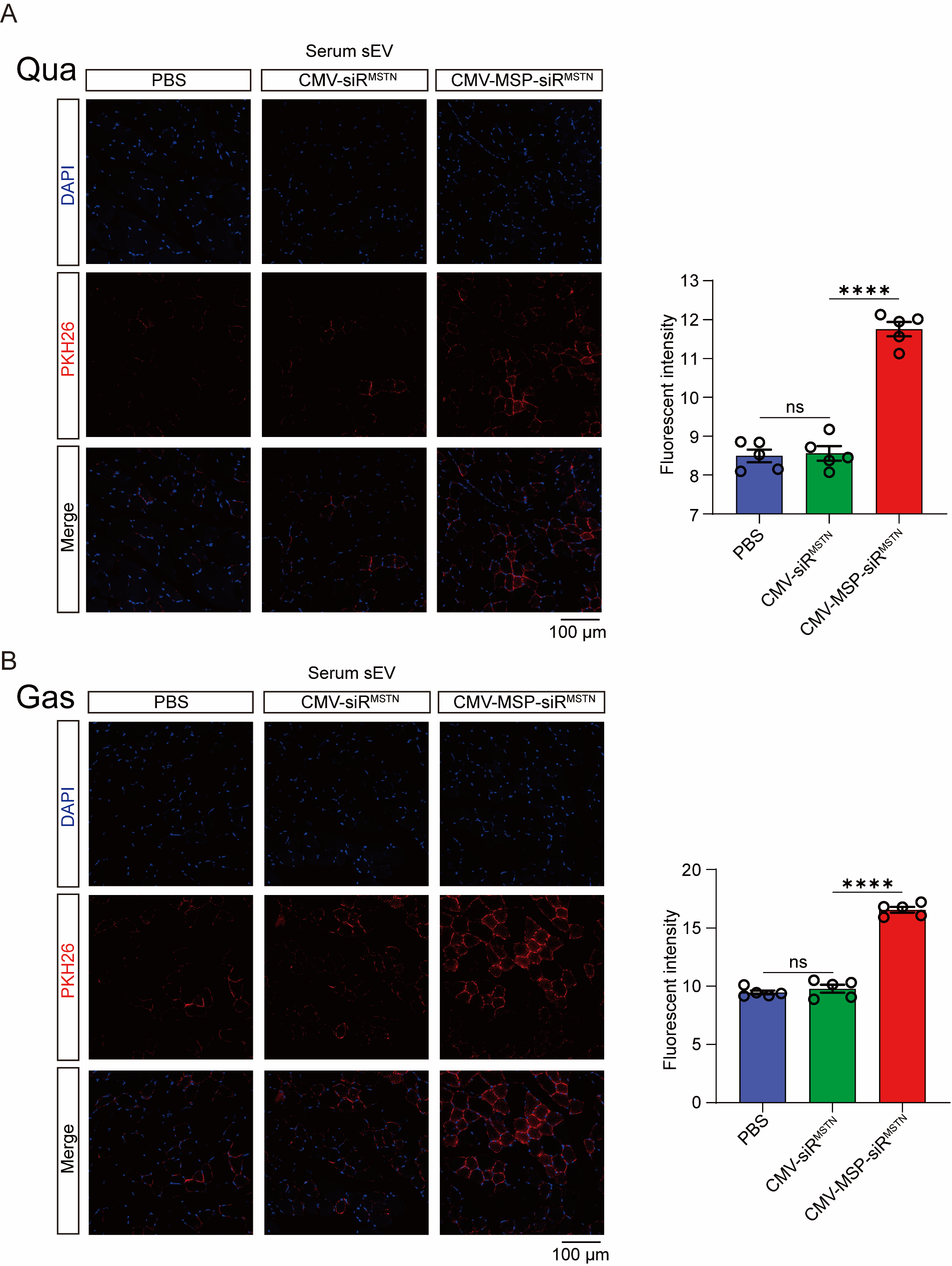


**Figure S3.** **Tracking the delivery of self-assembled sEVs to skeletal muscle.**

C57BL/6J mice were intravenously injected with PBS, CMV-siR^MSTN^, or CMV-MSP-siR^MSTN^ every 2 days for a total of seven injections. Nine hours after the final injection, serum sEVs were purified, labeled with PKH26 dye, and subsequently administered via tail vein injection into new recipient mice. (A) Fluorescence signals were then analyzed in the quadriceps muscle (Qua) (n = 5). (B) Fluorescence signals were then analyzed in the gastrocnemius muscle (Gas) (n = 5). Data are presented as mean ± SEM. Statistical significance was determined using one-way ANOVA followed by Bonferroni's multiple comparisons test for panels A and B. ****P < 0.0001; ns = not significant.


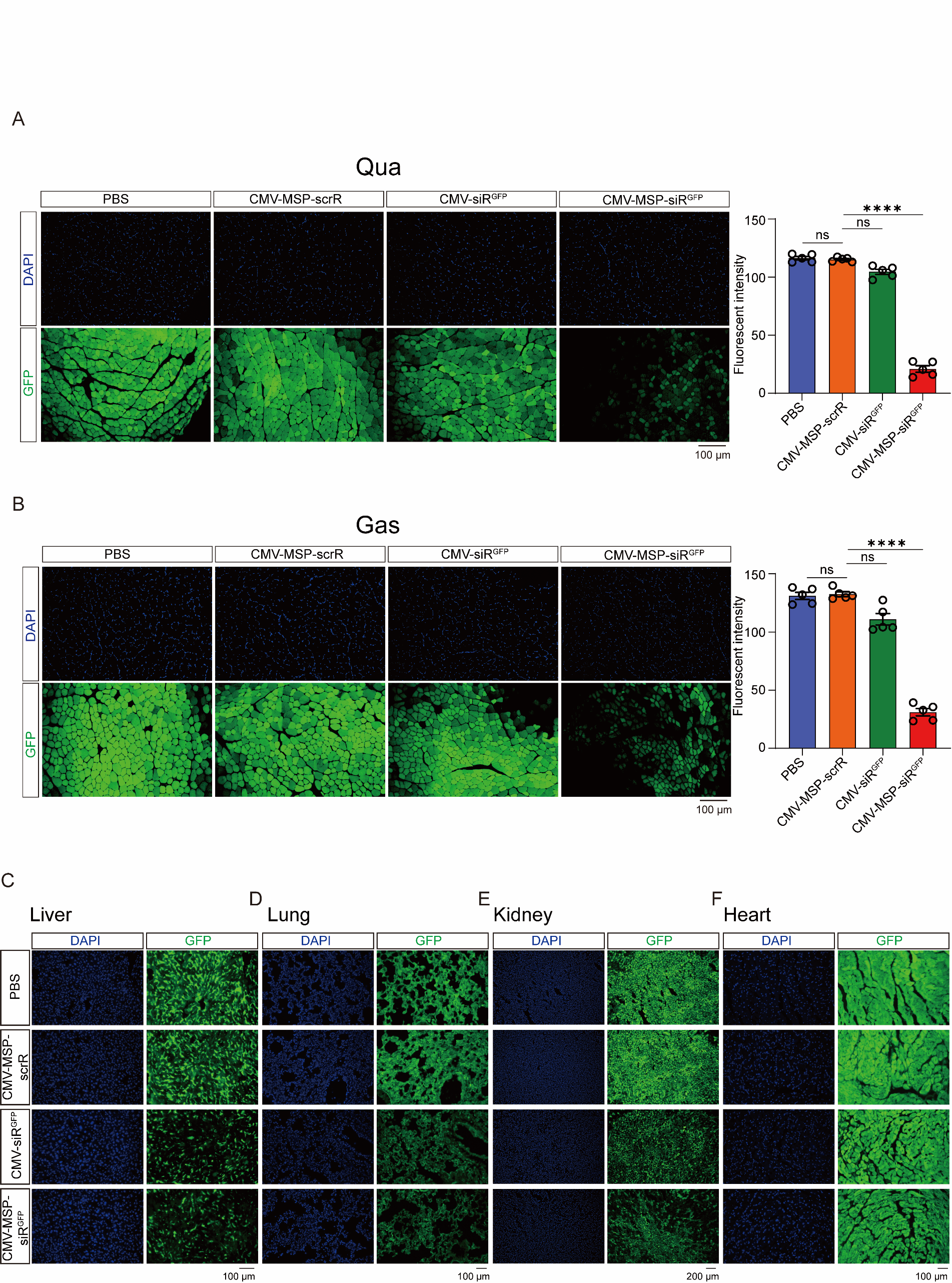


**Figure S4. Direct visualization of the suppression of GFP fluorescence levels *in vivo* by self-assembled GFP-siRNA.**

GFP-transgenic mice were intravenously injected with PBS or with 5 mg/kg of CMV-MSP-scrR, CMV-siR^GFP^, or CMV-MSP-siR^GFP^ constructs every 2 days for a total of seven doses. After the treatment period, mice were euthanized, and GFP fluorescence levels were assessed in frozen sections of the quadriceps (Qua), gastrocnemius (Gas), liver, lung, kidney, and heart. Representative fluorescence microscopy images of the quadriceps (A) (n = 5), gastrocnemius (B) (n = 5), liver (C), lung (D), kidney (E), and heart (F) are shown. GFP-positive signals are shown in green, and DAPI-stained nuclei are shown in blue. Scale bars are shown in each panel. Data are presented as mean ± SEM. Statistical significance was determined using one-way ANOVA followed by Bonferroni's multiple comparisons test for panels A and B. ****P < 0.0001; ns = not significant.


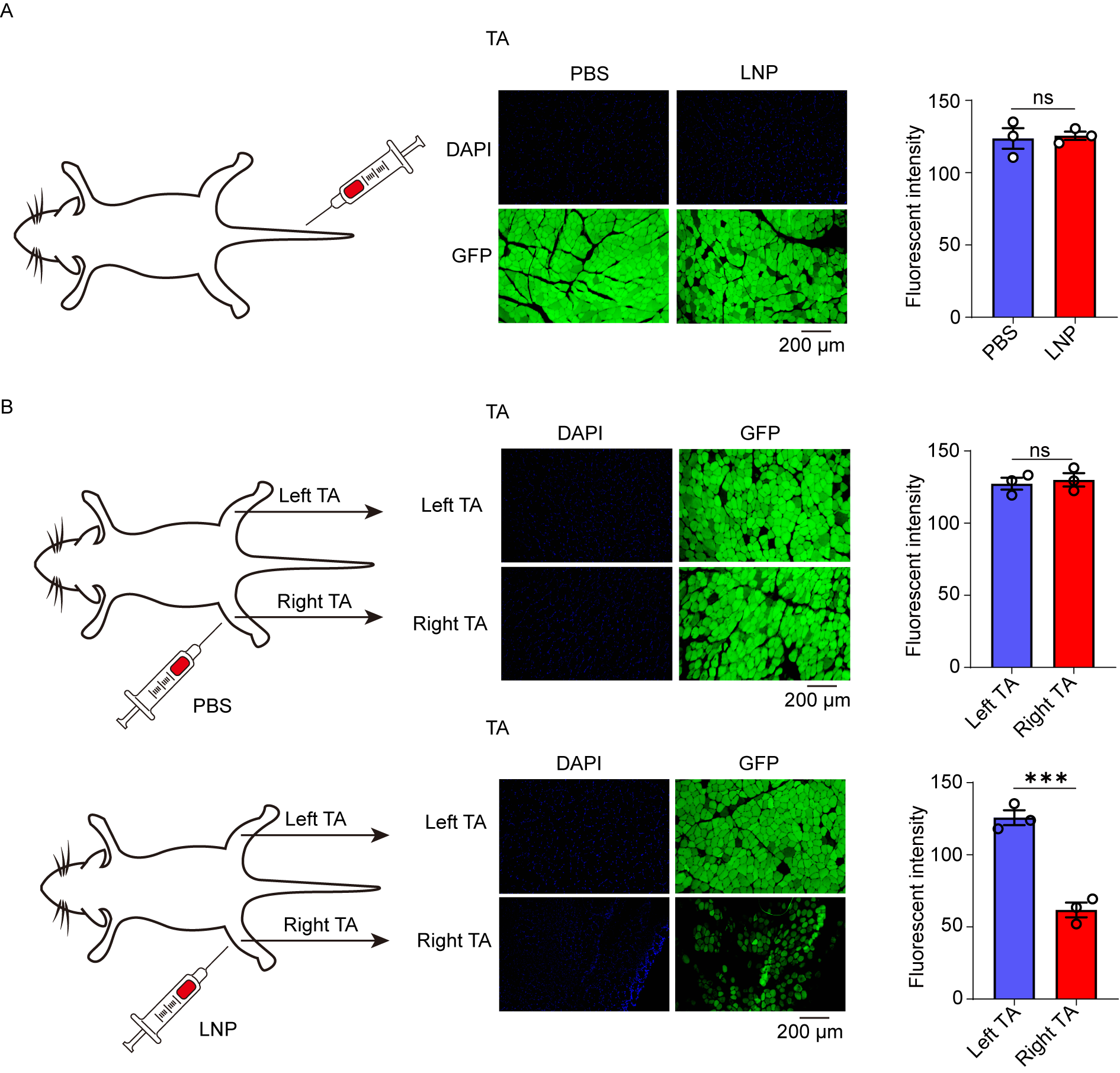


**Figure S5. Representative GFP fluorescence images of TA muscle following siRNA-LNP administration.**

(A) Systemic delivery via tail vein injection. Mice received GFP siRNA-LNPs or PBS control through the tail vein. GFP fluorescence (green) and DAPI-stained nuclei (blue) were visualized in TA muscles. (B) Local intramuscular delivery. Mice were administered GFP siRNA-LNPs or PBS directly into the right TA muscle, with the left TA serving as an internal control. Positive GFP signals are shown in green, and DAPI-stained nuclei are shown in blue. Scale bar = 200 μm. Data are presented as mean ± SEM. Statistical significance was determined using one-way ANOVA followed by Bonferroni's multiple comparisons test for panels A and B. ***P < 0.001; ns = not significant.


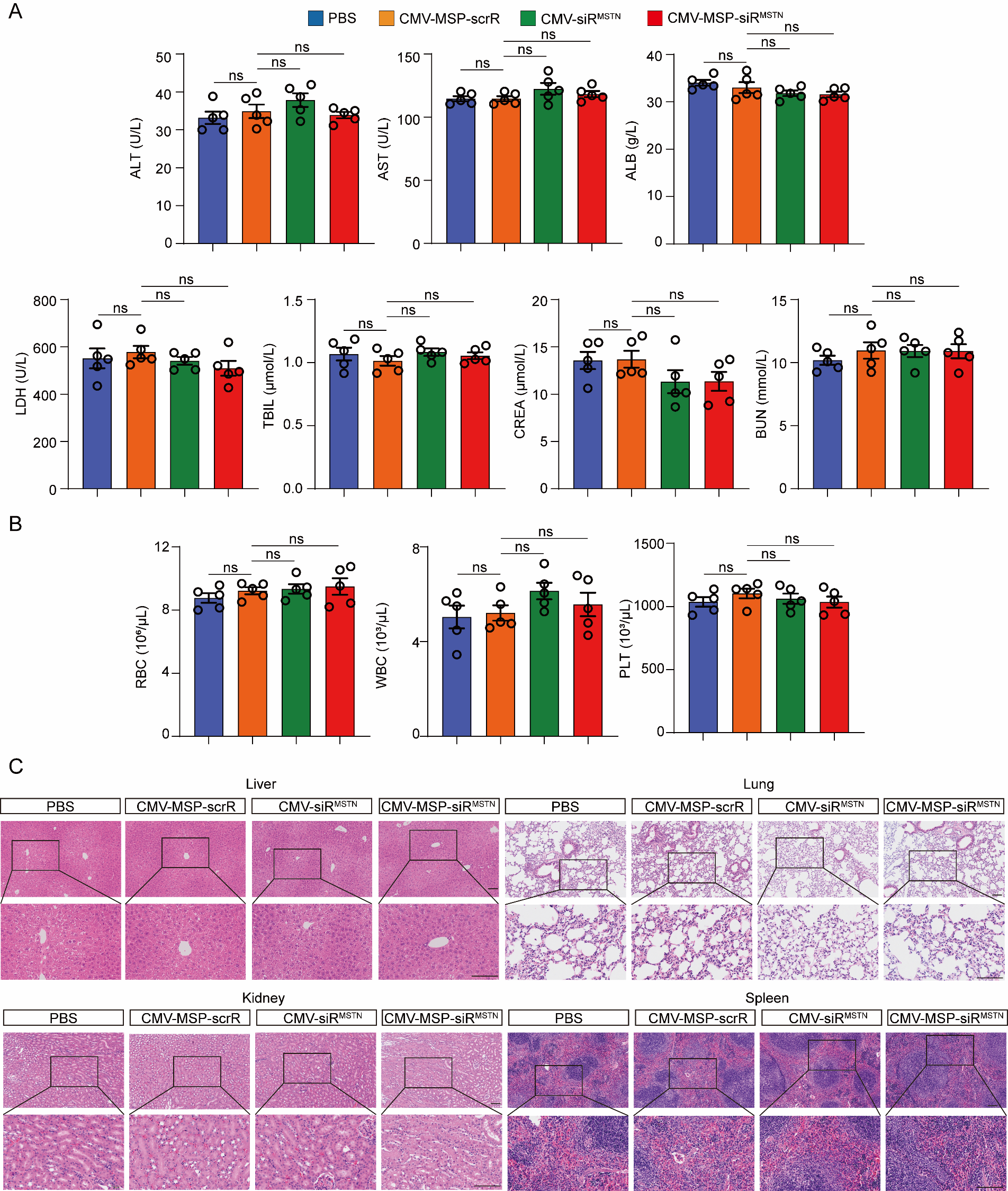


**Figure S6. Evaluation of toxic effects in mice following intravenous injection of synthetic constructs.**

C57BL/6 J mice were intravenously injected with PBS or with 5 mg/kg CMV-MSP-scrR, CMV-siR^MSTN^ or CMV-MSP-siR^MSTN^ constructs every 2 days for a total of seven times. After treatment, mice were sacrificed, and blood and tissue samples were collected and analyzed for serum biochemical indicators and tissue damage. (A) Serum biochemical indexes (ALT, AST, ALP, LDH, TBIL, CREA and BUN) (n = 5). (B) Counts of RBCs, WBCs and PLTs in peripheral blood (n = 5).

(C) Histological examination of the livers, lungs, kidneys and spleens of synthetic constructs-treated mice. Scale bar: 100 μm. Values are presented as mean ± SEM. Significance was determined using one-way ANOVA followed by Bonferroni's multiple comparisons in A and B. ns, not significant.

**
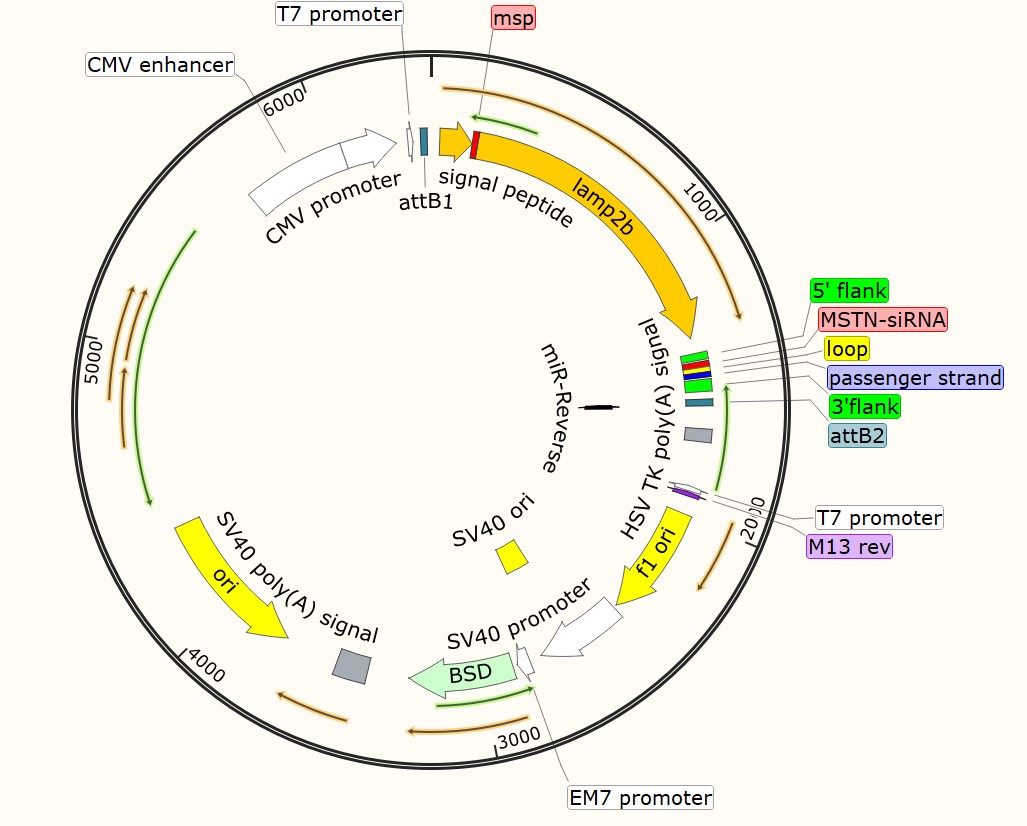
**

**Figure S7.** The scaffold and map of the CMV-MSP-siR^MSTN^ construct.

**Table S1. List of inserted sequences into synthetic construct**

|  | **DNA sequence** | **Mature Sequence** |
| --- | --- | --- |
| siRNA-1 | TAATGATTGTTTCCGTGGTAGGTTTTGGCCACTGACTGACCTACCACGAACAATCATTA | UAAUGAUUGUUUCCGUGGUAG |
| siRNA-2 | TAGAAAGTCAGACTCTGTAGGGTTTTGGCCACTGACTGACCCTACAGACTGACTTTCTA | UAGAAAGUCAGACUCUGUAGG |
| siRNA-3 | AACATTTGGGCTTGCCATCCGGTTTTGGCCACTGACTGACCGGATGGCGCCCAAATGTT | AACAUUUGGGCUUGCCAUCCG |
| siRNA-4 | TCAATACTCTGCCAAATACCAGTTTTGGCCACTGACTGACTGGTATTTCAGAGTATTGA | UCAAUACUCUGCCAAAUACCA |
|  |  | **Amino acid sequence** |
| MSP | GCCAGCAGCCTGAACATCGCC | ASSLNIA |

**Table S2. List of primer sequences**

| **mRNA primers** | **Orientation** | **Sequence (5’ to 3’)** |
| --- | --- | --- |
| MSTN | Fw | AGTGGATCTAAATGAGGGCAGT |
|  | Rv | GTTTCCAGGCGCAGCTTAC |
| Fbxo32 | Fw | CAGCTTCGTGAGCGACCTC |
|  | Rv | GGCAGTCGAGAAGTCCAGTC |
| MuRF1 | Fw | GTGTGAGGTGCCTACTTGCTC |
|  | Rv | GCTCAGTCTTCTGTCCTTGGA |
| GAPDH | Fw | AGGTCGGTGTGAACGGATTTG |
|  | Rv | TGTAGACCATGTAGTTGAGGTCA |
| **miRNA primers** | **Orientation** | **Sequence (5’ to 3’)** |
| siRNA-1 | Stem-loop | GTCGTATCCAGTGCAGGGTCCGAGGTATTCGCACTGGATACGACCTACCA |
|  | Fw | GCGCGTAATGATTGTTTCCG |
| siRNA-2 | Stem-loop | GTCGTATCCAGTGCAGGGTCCGAGGTATTCGCACTGGATACGACCCTACA |
|  | Fw | CGCGCGTAGAAAGTCAGACTC |
| siRNA-3 | Stem-loop | GTCGTATCCAGTGCAGGGTCCGAGGTATTCGCACTGGATACGACCGGATG |
|  | Fw | GCGAACATTTGGGCTTGC |
| siRNA-4 | Stem-loop | GTCGTATCCAGTGCAGGGTCCGAGGTATTCGCACTGGATACGACTGGTAT |
|  | Fw | GCGCGTCAATACTCTGCCAA |
| **Taqman Probe** | **Catalog** |  |
| siRNA-4 | Catalog:4398987 |  |
